# Examining the relationship between healthcare practitioners’ communication and patient adherence to treatment: a systematic review

**DOI:** 10.1186/s12913-026-14359-8

**Published:** 2026-03-25

**Authors:** Leila Keshtkar, Sarah Ahmed, Shaan Mohan, Harsh Kulshrestha, Keith Nockels, Cathy Harrell, Patrick Highton, Jeremy Howick

**Affiliations:** 1 Stoneygate Centre for Empathic Healthcare, Leicester Medical School, University of Leicester, George Davies Centre, Lancaster Rd, Leicester, LE1 7HA UK; 2 Library and Learning Services, University of Leicester (Until July 2025), David Wilson Library, Leicester, LE1 7RH UK; 3 Diabetes Research Centre, University of Leicester, Leicester General Hospital, Leicester, LE5 4PW UK

**Keywords:** Communication, Patient adherence, Healthcare intervention

## Abstract

**Background:**

Effective communication between healthcare providers and patients is essential to ensure that patients understand and follow their treatment plans. This study examined the relationship between patient-practitioner communication and patient adherence to healthcare interventions.

**Methods:**

Ovid MEDLINE, CINAHL, APA PsycInfo, CENTRAL, and Scopus were searched from 2014 to January 2025. Reference lists of eligible articles were manually searched. Studies in any language that quantified the effects of patient–practitioner communication on adherence were included. Two independent reviewers extracted data, assessed the risk of bias, and appraised the strength of evidence. Risk of bias was assessed using RoB2 for randomised controlled trials (RCTs) and the Joanna Briggs Institute (JBI) for observational studies.

**Results:**

Twenty-four eligible studies (8,671 patients) were included: six RCTs (733 patients) and 18 observational studies (7,938 patients). Among the observational studies, 17 were cross-sectional (14 high quality and three moderate quality), and one was a high-quality retrospective cohort study. Of the six RCTs, four had some concerns, and two had a high risk of bias. Pooled analysis of the RCTs demonstrated a small, non-significant positive effect of the communication intervention on adherence (standardised mean difference: 0.42, 95% CI: − 0.08 to 0.93; *p* = 0.10). However, heterogeneity was high (I² = 88%), and the certainty of evidence was very low. All observational studies suggested a positive association between effective practitioner communication and improved treatment adherence.

**Conclusion:**

Enhanced practitioner communication skills are associated with better adherence. Interventions targeting practitioner communication skills may represent a practical and impactful approach to support patients in following their healthcare recommendations.

**Supplementary Information:**

The online version contains supplementary material available at 10.1186/s12913-026-14359-8.

## Introduction

Patient adherence is defined as “the extent to which a person’s behaviour–taking medication, following a diet, and/or executing lifestyle changes, corresponds with agreed recommendations from a healthcare provider” [1]. Although adherence plays an important role in patient outcomes [2–4], approximately 50% of patients do not take their long-term treatment for chronic conditions as prescribed [5, 6]. A 2018 systematic review examining primary nonadherence for the most commonly prescribed chronic disease medications found that, on average, 15 of every 100 medications prescribed for chronic conditions are not filled by patients [7]. Another systematic review similarly revealed that a substantial proportion of patients do not adequately adhere to prescribed cardiovascular medications [8]. Low adherence to healthcare interventions also imposes a considerable financial burden [5]. For example, in 2022, the International Longevity Centre (ILC) [9] reported that €125 billion is lost each year across Europe due to non-adherence to medication. In England, the estimated opportunity cost of missed health gains due to nonadherence exceeds £930 million annually across just five diseases (asthma, type 2 diabetes, high cholesterol/coronary heart disease, hypertension, and schizophrenia) [10].

Interventions to improve adherence are complex and multifaceted and can include, for example, medication reminders and patient education programs [11, 12]. However, improving patient-practitioner communication plays a key role in patient adherence [13]. In 2003, the World Health Organisation (WHO) [6] provided comprehensive guidance on the importance of adherence in the management of chronic diseases. The report emphasised the importance of adherence to long-term therapies in improving health outcomes and reducing the burden of chronic diseases. It also highlighted that many health professionals lack the communication skills necessary to effectively support patient adherence. In 2009, the National Institute for Health and Care Excellence [14] issued guidance emphasising the need to improve communication between healthcare practitioners and patients to support medication adherence. The guidance encourages healthcare professionals to actively engage with patients to identify and address barriers to adherence. Similarly, NHS England’s 2016 report, “A Long and Winding Road” [15], highlighted that effective communication is a critical factor in improving patient adherence. The report recommends strategies such as training healthcare professionals in communication skills and ensuring that patients receive clear and understandable information about their treatments. Relatedly, better communication can contribute to patients’ understanding of illness, the risks and benefits of treatment, and enhance adherence [2]. Several studies also suggest that practitioners who demonstrate strong communication competencies, such as encouraging patients to express concerns without interruption, using open-ended inquiry, employing active listening and understanding patient feelings, can foster better patient engagement and trust [16, 17]. These competencies are foundational to mutuality and partnership within the therapeutic relationship and have been linked to improved adherence and more positive health outcomes [18]. Shared decision-making frameworks further highlight the importance of collaborative dialogue in aligning treatment plans with patients’ values and preferences [19]. In addition, DiMatteo et al. [20] highlight the central role of patient-practitioner communication in promoting treatment adherence, characterising the therapeutic relationship as a dynamic process shaped by trust, respect, and perceived clinician support. These communicative processes may be particularly salient in the context of evolving healthcare delivery models, where clinical encounters are often brief or digitally mediated, making effective communication essential for maintaining care relationships and supporting adherence.

Several systematic reviews have investigated the relationship between communication and patient adherence [2, 13, 21, 22]. However, these reviews have been limited to specific healthcare disciplines (such as physicians) [13] or particular forms of communication, such as digital communication for patient medication adherence [21–23]. Moreover, several studies have been published [24, 25] in recent years, but these new studies have not yet been reviewed and synthesised. To address these limitations, the present review adopts a broader perspective across healthcare practitioners. Although practitioners differ in training, scope of practice, and the nature of their interactions with patients, they share core communication behaviours relevant to adherence—such as information provision, clarification, and empathy—across healthcare disciplines. However, the way these behaviours are delivered may vary by practitioner type and clinical context. Therefore, this review does not assume equivalence across practitioner types; rather, practitioner-specific differences are explored through stratified synthesis.

This study aimed to systematically identify, appraise, and synthesise the evidence regarding the relationship between patient (or carer)-practitioner communication and patient adherence to healthcare interventions.

## Methods

This protocol has been described using the Preferred Reporting Items for Systematic Reviews and Meta-Analyses Protocols (PRISMA-P) [26] and registered in PROSPERO (CRD42025616150).

### Eligibility criteria

Our inclusion criteria are summarised in Supplementary Tables 1, and the definition of key terms is provided in Supplementary File 1. We included any study that quantitatively reported a relationship between patient (or carer)-practitioner communication and patient adherence to healthcare interventions.

We included studies involving patients with both chronic and acute conditions, of any age, who were treated by any healthcare practitioner (e.g., physicians, nursing, etc.). We included any setting, as long as the communication was between the patient (or carer) and practitioner.

The communication could involve live conversations, encompassing both face-to-face communication and communication via digital media (including telephone, mobile phone, and video). Effective patient-practitioner communication is essential for improving adherence, as it helps build trust, address patient concerns, and motivate behaviour change. Therefore, we included patient-practitioner communication aimed specifically at improving adherence as well as general patient-practitioner communication that is not specifically aimed at improving adherence (such as counselling and empathic behaviour). We excluded electronic reminders, recorded messages and voicemails, emails, short message service (SMS) reminders, written instructions, and leaflets.

For studies that had a control group, the comparator is usual care or a less comprehensive communication approach provided as part of standard care. For studies without a control group, there is no comparator.

The primary outcome is patient adherence to healthcare interventions. We included studies that measured patient adherence using any method, such as self-reports [27], wearable devices to track physical activity levels [28], medical chart records and appointment records [29], prescription records, pharmacy refill data, pill counts, or medication event monitoring system pill bottle caps [30–32]. We included articles reported in any language.

### Data source and searches

The search strategy is provided in Supplementary File 2. We searched the following databases from 2014 to 23 January 2025: MEDLINE, CINAHL, APA PsychINfo, CENTRAL, and Scopus. The cut-off date of 2014 was chosen to reflect current rather than historical practice [33]. By this time, earlier systematic reviews [13, 22] had already synthesised existing research on communication and adherence, allowing us to focus on more recent developments rather than replicate previous work. This timeframe is further justified by substantial changes in healthcare delivery over the past decade, including an increased emphasis on communication-focused interventions to improve care delivery and enhance quality of care [15, 34]. We also manually searched the reference lists of included studies to identify additional relevant studies [6, 14]. The search strategy was peer-reviewed using the Peer Review of Electronic Search Strategies (PRESS) checklist [35] (see Supplementary File 3).

### Study selection

Records were imported into Covidence. Two reviewers (from among LK, SA, SM, HK, and PH) independently screened titles, abstracts, and full texts for eligibility. Data from the eligible full texts were extracted using the Covidence extraction form. Discrepancies in screening or extraction were resolved by discussion, if necessary, with a third reviewer (JH).

### Data extraction and quality assessment

Two reviewers (from among LK, SA, SM, HK, and PH) independently summarised and recorded data from the included studies. Extracted data included: study details (including first author, lead author contact details, date published, country of study), study aim, study design, participant demographics (e.g., age, ethnicity, sex), demographics of healthcare staff, details of intervention, patients, healthcare setting, how communication and patient adherence were measured, comparator, and outcomes details. Missing or unclear information was addressed by contacting the study authors for clarification.

Two authors independently assessed the risk of bias using the Cochrane risk-of-bias tool for randomised trials (RoB2) [36] and relevant Joanna-Briggs Institute (JBI) checklists [37]. Disagreements were resolved by discussion or consultation with a third author (JH). The RoB2 tool assesses risk of bias in randomised trials across five domains—randomisation, deviations from intended interventions, missing outcome data, outcome measurement, and selection of reported results—each study rated as low risk, some concerns, or high risk. For JBI, each item was rated as yes, no, unclear, or not applicable, with two points assigned for yes, one point for unclear, and zero points for no. The overall quality appraisal was categorised as follows: high quality (> 7 points), moderate quality (≤ 7 to > 5 points), or low quality (≤ 5 points) [38]. We used the GRADE (Grading of Recommendations, Assessment, Development, and Evaluations) [39] to evaluate the overall certainty of evidence for all outcomes, ranging from very low to high.

### Data synthesis and analysis

Due to the substantial clinical and methodological heterogeneity, as well as diversity in reported effect measures—such as odds ratios, linear regression coefficients, and correlation coefficients—a single meta-analysis across all included studies was not considered appropriate [40].

Randomised controlled trials (RCTs) were pooled using standardised mean differences (SMDs) as the summary effect measure under a random-effects model. In addition, a random-effects meta-analysis was conducted for cross-sectional studies reporting Pearson correlation coefficients (r) directly, as r represents a common and directly comparable measure of association.

Studies that were not eligible for quantitative synthesis were synthesised narratively following the synthesis without meta-analysis (SWiM) reporting guidelines [41].

### Patient and public involvement

One patient representative (CH) was a co-author of the study. CH reviewed the protocol and suggested improvements, including clarifying the wording of patient-reported outcomes and ensuring the selected outcomes were meaningful to patients. These suggestions were incorporated into the final protocol prior to study initiation.

## Results

Our search identified 25,156 records. After excluding duplicates, 18,761 articles were screened by title and abstract according to our exclusion criteria, with 18,706 eliminated and a further 32 excluded after full-text review. Twenty-four studies were included (see PRISMA flow chart, Supplementary Fig. 1).

### Characteristics of included studies

Table 1 summarises the key details of the included studies. Twenty-four studies reported a total number of 8,671 patients from 16 [42] to 2,571 [43] (median = 267). Eight studies were completed in Asia [18, 44–50], 11 studies in North America [42, 43, 51–59], three studies in Africa [60–62], and two in Europe [24, 25]. The types of healthcare staff were pharmacists in four studies [24, 42, 53, 58], hospital physicians in nine studies [25, 44–46, 48, 50, 52, 61, 62], nurses in one [47], primary care physicians in four [18, 43, 56, 60], and not specified in six studies [49, 51, 54, 55, 57, 59]. Eight studies were conducted in hospitals [42, 44–46, 48, 50, 61, 62], three in outpatient clinics in hospitals [25, 47, 52], four in primary healthcare centres [18, 43, 51, 60], three in pharmacies [24, 53, 58], one in a post-deployment health clinic [56], one in a psychiatry department of a tertiary healthcare centre [49], and not reported in four studies [54, 55, 57, 59].

There were six RCTs [24, 42, 47, 52, 53, 58], one retrospective cohort study [43], and the rest of the studies were cross-sectional [18, 25, 44–46, 48–51, 54–57, 59–62]. The interventions named empathic behaviour in two studies [18, 46], pharmacist discharge counselling [42], pharmacist telephone calls [58], motivational interviewing in two studies [47, 53], and patient-practitioner communication in the remaining studies [24, 25, 43–45, 48–52, 54–57, 59–62].

The studies used different scales and tools to assess communication, such as the Consultation and Relational Empathy (CARE) measurement tool [63], Communication Assessment Tool [64], Patient-Centred Communication (65), and the Doctor-Patient Communication Questionnaire [66] (see Supplementary Table 2). Nineteen studies reported medication adherence as an outcome [18, 24, 25, 42, 43, 45, 47–51, 53–55, 58–62], while five studies measured overall adherence to medical advice [44, 46, 52, 56, 57]. In three studies, patient adherence was measured using medical chart records [46, 52, 58]. Eighteen relied on patient self-report [18, 25, 42, 44, 45, 47–51, 54–57, 59–62], two used pharmacy refill data [24, 43], and one employed a multimethod approach (pharmacy verification and self-report) [53]. Among self-report studies, a range of validated scales were used, most commonly the Morisky Medication Adherence Scale [67], the Diabetes Self-Management Questionnaire [68], the Medication Adherence Rating Scale [69], and the Medication Adherence Report Scale [70] (see Supplementary Table 3).

### Risk-of-bias results

Fourteen cross-sectional studies [18, 25, 44–46, 48–51, 54, 55, 60–62] and one retrospective cohort study [43] had high quality. Three cross-sectional studies [56, 57, 59] had moderate quality. Four RCTs had some concerns [24, 47, 52, 53], and two had a high risk of bias [42, 58] (see Supplementary Table 4).

The main reasons for concerns regarding risk of bias in the RCTs were methodological limitations, including self-reported outcomes, lack of blinding, and unclear randomisation procedures. Studies were rated as high risk due to reliance on self-reported data, inadequate reporting of attrition, incomplete outcome data, and potential crossover effects. High attrition in small samples further compromised outcome validity.

Similarly, concerns about bias in the cross-sectional studies primarily stemmed from unclear or poorly defined eligibility criteria, increasing the risk of selection bias. Across these studies, exposure and outcome measures were largely self-reported, limiting the reliability and objectivity of the findings. Additional concerns arose from vague definitions, insufficient identification and handling of confounding variables.

**Table 1:** Details of included studies

Study ID	Risk of bias RoB2 (low, some concerns, high) JBI (high, moderate, low quality)	Study design	Patient type (*N*)	Effect size	Type of healthcare providers	Setting	Intervention	Modalities of communication	Outcome	Measurement strategies	Continent/country
Ibrahim 2020 [46]	High quality	Cross-sectional	Diabetic patients (214)	Odds ratio (OR) = 1.05	Hospital physicians	Hospital/ Secondary care	Empathic behaviour	Face-to-face	Overall adherence to medical advice	Medical chart records	Asia/ Saudi Arabia
Çakmak 2021 [47]	Some concerns	RCT	Cancer patients (80 Intervention: 40, Control: 40)	Intervention: Mean (SD) 83.0 (5.03) Control: Mean (SD) 68.1 (10.68)	Nurse	Outpatient clinic in a hospital/ Secondary care	Motivational interviewing	Face-to-face & telephone call	Medication adherence	Self-report	Asia/ Turkey
Vinluan 2015 [42]	High	RCT	Heart failure patients (16 Intervention: 7, Control: 9)	Intervention: Mean (SD) 6.6 (1.80) Control: Mean (SD) 6.1 (1.70)	Pharmacist	Hospital/ Secondary care	Pharmacist discharge counselling	Face-to-face & telephone call	Medication adherence	Self-report	North America/ US
Lee 2017 [51]	High quality	Cross-sectional	Hypertensive patients (191)	Standardised beta (β) = 0.25	Not specified	Community centre/ Primary care	Patient-practitioner communication	Face-to-face	Medication adherence	Self-report	North America/ US
Eltaher 2020 [18]	High quality	Cross-sectional	Diabetic patients (391)	*r* = − 0.40	Primary care physicians	Primary healthcare centre/ Primary care	Empathic behaviour	Face-to-face	Medication adherence	Self-report	Asia/ Saudi Arabia
Fosu 2022 [60]	High quality	Cross-sectional	HIV patients (349)	β = 0.38	Primary care physicians	Community centre/ Primary care	Patient-practitioner communication	Face-to-face	Medication adherence	Self-report	Africa/ Ghana
Burmeister 2023 [52]	Some concerns	RCT	Cancer patients (44 Intervention: 23, Control: 21)	Intervention: Mean (SD) 93.95 (10.73) Control: Mean (SD) 95.82 (7.69)	Hospital physicians	Outpatient clinic in a hospital/ Secondary care	Patient-practitioner communication	Face-to-face	Overall adherence to medical advice	Medical chart records	North America/ US
Teymuri 2023 [48]	High quality	Cross-sectional	Hospitalised patients (284)	β = 0.33	Hospital physicians	Hospital/ Secondary care	Patient-practitioner communication	Face-to-face	Medication adherence	Self-report	Asia/Iran
Nwosu 2023 [61]	High quality	Cross-sectional	General outpatient (213)	*r* = 0.32	Hospital physicians	Hospital/ Secondary care	Patient-practitioner communication	Face-to-face	Medication adherence	Self-report	Africa/ Nigeria
Eyler 2016 [53]	Some concerns	RCT	Patients admitted with pneumonia (30 Intervention: 16, Control: 14)	Intervention: 87% Control: 64%	Pharmacist	Pharmacy/ Primary care	Motivational interviewing	Face-to-face	Medication adherence	Pharmacy verification and self-report	North America/ US
Michiels 2019 [24]	Some concerns	RCT	Diabetic patients (377 Intervention: 189, Control: 188)	Intervention: Mean (SD) 94.8 (11) Control: Mean (SD) 92.3 (15)	Pharmacist	Pharmacy/ Primary care	Pharmaceutical interview	Face-to-face	Medication adherence	Pharmacy refill data	Europe/ France
Swain 2015 [44]	High quality	Cross-sectional	Patients with hypertension (300)	β = 0.24	Hospital physicians	Hospital/ Secondary care	Patient-practitioner communication	Face-to-face	Overall adherence to medical advice	Self-report	Asia/ India
Lampert 2022 [54]	High quality	Cross-sectional	Not reported (399)	*r* = 0.14	Not specified	Not reported	Patient-practitioner communication	Face-to-face	Medication adherence	Self-report	North America/ US
Ocansey 2024 [62]	High quality	Cross-sectional	Schizophrenia patients (117)	β = 0.45	Hospital physicians	Hospital/ Secondary care	Patient-practitioner communication	Not reported	Medication adherence	Self-report	Africa/ Ghana
Young 2017 [55]	High quality	Cross-sectional	Asthma patients (452)	*r* = 0.27	Not specified	Not reported	Patient-practitioner communication	Not reported	Medication adherence	Self-report	North America/ US
Phillips 2017 [56]	Moderate quality	Cross-sectional	Chronic disease patients (204)	*r* = 0.41	Primary care physicians	Post-deployment health clinic / Tertiary care	Patient-practitioner communication	Face-to-face	Overall adherence to medical advice	Self-report	North America/ US
Świątoniowska-Lonc 2020 [25]	High quality	Cross-sectional	Patients with hypertension (250)	*r* = − 0.29	Hospital physicians	Outpatient clinic in a hospital/ Secondary care	Patient-practitioner communication	Face-to-face	Medication adherence	Self-report	Europe/ Poland
Chang 2021 [43]	High quality	Retrospective cohort	Patients with hypertension (2571)	OR = 1.38	Primary care physicians	Primary healthcare centre/ Primary care	Patient-practitioner communication	Not reported	Medication adherence	Pharmacy refill data	North America/ US
Soyoon 2022 [57]	Moderate quality	Cross-sectional	Diabetic patients (317)	*r* = 0.54	Not specified	Not reported	Patient-practitioner communication	Not reported	Overall adherence to medical advice	Self-report	North America/ US
Du 2020 [45]	High quality	Cross-sectional	Outpatients with pulmonary tuberculosis (564)	Estimate (b) = 0.21	Hospital physicians	Hospital/ Secondary care	Patient-practitioner communication	Not reported	Medication adherence	Self-report	Asia/ China
Mercadante 2021 [58]	High	RCT	Not reported (186 Intervention: 97, Control: 89)	Intervention: Mean (SD) 0.71 (0.23) Control: Mean (SD) 0.73 (0.25)	Pharmacist	Pharmacy/ Primary care	Pharmacist call	Telephone call	Medication adherence	Medical chart records	North America/ US
Ghosh 2022 [49]	High quality	Cross-sectional	Patients with severe mental disorder (152)	*r* = 0.38	Not specified	Psychiatry centre/ Tertiary care	Patient-practitioner communication	Not reported	Medication adherence	Self-report	Asia/ India
Trepka 2023 [59]	Moderate quality	Cross-sectional	HIV patients (560)	OR = 1.99	Not specified	Not reported	Patient-practitioner communication	Not reported	Medication adherence	Self-report	North America/ US
Barakat 2024 [50]	High quality	Cross-sectional	Patients with dyslipidaemia (410)	Unstandardised B = 0.16	Hospital physicians	Hospital/ Secondary care	Patient-practitioner communication	Face-to-face	Medication adherence	Self-report	Asia/Jordan

### Evidence synthesis

Of the 24 studies included, six were RCTs [24, 42, 47, 52, 53, 58]. These RCTs assessed adherence-related outcomes using different measurement instruments and data sources, including self-report adherence scales, pharmacy refill data, and medical chart records. Given the use of different adherence measurement scales across trials, the RCTs were pooled in a random-effect meta-analysis using standardised mean differences (SMD) as the summary effect measure. The pooled analysis demonstrated a small, non-significant effect in favour of the intervention (SMD = 0.42, 95% CI − 0.08 to 0.93, *p* = 0.10). Heterogeneity across trials was high (I² = 88%, τ² = 0.31, *p* < 0.001). The certainty of evidence was very low (see Supplementary Table 5). After excluding two studies [42, 58] with high risk of bias, the effect measure remained statistically non-significant, and heterogeneity increased from 88% to 91%.

Eight cross-sectional studies [18, 25, 49, 54–57, 61] reporting Pearson correlation coefficients (r) were pooled and suggested a moderate positive association between practitioner communication and adherence (pooled *r* = 0.35, 95% CI 0.25 to 0.44). However, heterogeneity was substantial (I² = 85.5%, *p* < 0.001) (see Supplementary Fig. 2).

Heterogeneity across studies may have been attributable to differences in patient populations (e.g., diabetes, hypertension, asthma, and mental health conditions), clinical settings (e.g., primary, secondary, and tertiary care), types of healthcare providers (e.g., hospital/primary care physicians and pharmacists), outcome definitions (medication adherence versus overall adherence to medical advice) and geographical regions (e.g., Asia, Africa, North America and Europe).

Table 2 summarises the evidence synthesis results.

**Table 2:** Evidence synthesis results

Outcome	No. studies (no. patients)	Results
**By outcome**		
Overall adherence	Four cross-sectional studies (1035)	A statistically significant association between practitioners’ empathy and overall adherence (OR = 1.05, 95% CI 1.00 to 1.10, *p* = 0.04).
		A significant association between communication and overall adherence (β = 0.24, *p* < 0.01).
		Positive correlation between communication and overall adherence (r = 0.41).
		Positive correlation between communication and overall adherence (r = 0.54).
	One RCT (44)	No significant difference in patient treatment adherence (mean difference: −1.8, 95% CI − 7.5 to 3.8; *p* = 0.51).
Medication adherence	Five RCTs (744)	A small, non-significant positive effect of communication on medication adherence (SMD = 0.53, 95% CI − 0.05 to 1.11, *p* = 0.07).
	Six cross-sectional studies (1857)	Significant positive association between communication and medication adherence (Pooled *r* = 0.30, 95% CI 0.21 to 0.38).
	Seven cross-sectional studies and one retrospective cohort study (5046)	All reported a positive association between communication and medication adherence.
**By type of healthcare practitioner**		
Pharmacist	Four RCTs (664)	No statistically significant effect of the interventions on medication adherence (SMD = 0.13, 95% CI − 0.08 to 0.33, *p* = 0.24).
Hospital physician	Eight cross-sectional studies (2352)	A statistically significant association between physicians’ empathy and overall adherence to treatment (OR = 1.05, 95% CI 1.00 to 1.10, *p* = 0.04).
		A statistically significant association between physician communication and overall adherence to treatment (β = 0.24, *p* < 0.01).
		Hospital physicians’ communication positively influenced medication adherence (β = 0.33, *p* < 0.001).
		A significant positive correlation between perceived physician communication and medication adherence (β = 0.45, 95% CI 0.13 to 0.43, *p* < 0.001).
		Better medication adherence was associated with improved hospital physicians’ communication (B = 0.16, *p* = 0.003).
		Hospital physicians’ communication was positively related to medication adherence (Est.=0.21, *p* < 0.001).
		A significant and positive correlation between perceived communication and medication adherence (*r* = 0.31, *p* < 0.01).
		A statistically significant negative correlation between communication and medication adherence (*r* = − 0.29, *p* < 0.001).
	One RCT (44)	No significant difference in patient treatment adherence was observed (mean difference: −1.8, 95% CI − 7.5 to 3.8; *p* = 0.51).
Primary care physicians	Three cross-sectional studies and one retrospective cohort study (3515)	A statistically significant negative correlation was found between physicians’ empathy score and adherence score (*r* = − 0.40, *p* < 0.001).
		A strong positive association between primary care physicians’ communication and medication adherence (β = 0.38, 95% CI 0.09 to 0.18, *p* < 0.001).
		A significant association between communication and increased adherence to medications (OR = 1.38, 95% CI 1.14 to 1.67).
		Practitioner communication behaviours were positively associated with better treatment adherence (*r* = 0.41, *p* < 0.01).
Nurses	One RCT (80)	Communication-based nursing interventions significantly improved adherence among cancer patients compared with usual care (83.0 ± 5.03 vs. 68.1 ± 10.68, *p* < 0.001).

### Synthesis by outcome

#### Overall adherence to medical advice

Four cross-sectional studies [44, 46, 56, 57] with high quality [44, 46] and moderate quality [56, 57] examined the association between communication and overall adherence to medical advice. One study [46] reported a statistically significant association between practitioners’ empathy and overall adherence to treatment (OR = 1.05, 95% CI 1.00 to 1.10, *p* = 0.04). In another study [44], practitioner communication was significantly associated with overall adherence (β = 0.24, *p* < 0.01).

Furthermore, two other studies [56, 57] found a positive correlation between communication and overall adherence to healthcare intervention, with correlation coefficients of 0.41 and 0.54, respectively.

In contrast, one RCT [52] with some concerns about bias also found no statistically significant difference in treatment adherence between the intervention and control groups.

#### Medication adherence

Five RCTs [24, 42, 47, 53, 58] with some concerns about bias [24, 47, 53] and high risk of bias [42, 58] assessed the effect of communication on medication adherence. The pooled analysis showed a small, non-significant positive effect of the intervention on adherence (SMD = 0.53, 95% CI − 0.05 to 1.11, *p* = 0.07). However, substantial heterogeneity was present across studies (I² = 90%). The certainty of evidence was very low (see Supplementary Table 6).

Thirteen cross-sectional studies [18, 25, 45, 48–51, 54, 55, 59–62] and one retrospective cohort study [43] assessed the association between communication and medication adherence. Most studies were rated as high quality [18, 25, 43, 45, 48–51, 54, 55, 60–62], and one was rated as moderate quality [59].

Among these, six cross-sectional studies [18, 25, 49, 54, 55, 61] reporting correlation coefficients (r) were pooled, which showed a statistically significant positive association between practitioner communication and medication adherence (pooled *r* = 0.30, 95% CI 0.21 to 0.38). Moderate to substantial heterogeneity was observed (I² = 68.4%, *p* = 0.007).

The remaining eight studies [45, 48, 50, 51, 54, 59, 60, 62] that were not pooled each reported a positive association between effective communication and improved medication adherence.

### Synthesis by type of healthcare practitioner

Physicians, nurses, and pharmacists differ in professional training, scope of practice, and the nature and duration of their interactions with patients, which may influence treatment adherence through distinct mechanisms. Synthesising these provider groups may therefore obscure important differences across practitioner types. Accordingly, analyses were stratified by type of healthcare practitioner.

#### Pharmacists

Four RCTs [24, 42, 53, 58] with some concerns about bias [24, 53] and high risk of bias [42, 58] investigated the effect of pharmacist communication interventions on medication adherence. The pooled analysis showed no statistically significant effect of the interventions (SMD = 0.13, 95% CI − 0.08 to 0.33, *p* = 0.24), with low heterogeneity (I² = 22). The certainty of evidence was low (see Supplementary Table 7).

#### Hospital physicians

Two high-quality cross-sectional studies [44, 46] and one RCT [52] with some concerns about bias investigated the effect of hospital physicians’ communication on overall adherence to medical advice. One cross-sectional study [46] found a statistically significant association between physicians’ empathy and overall adherence to treatment (OR = 1.05, 95% CI 1.00 to 1.10, *p* = 0.04). Similarly, another study [44] reported a statistically significant association between physician communication and overall adherence (β = 0.24, *p* < 0.01).

However, one RCT [52] found that improvements in the hospital physicians’ communication with patients did not result in a statistically significant difference in treatment adherence.

Six high-quality cross-sectional studies [25, 45, 48, 50, 61, 62] examined the association between hospital physicians’ communication and medication adherence. One study [48] reported that hospital physicians’ communication was positively associated with medication adherence (β = 0.33, *p* < 0.001). Similarly, another study [62] found a significant positive association between perceived physician communication and medication adherence (β = 0.45, 95% CI 0.13 to 0.43, *p* < 0.001). One study [45] also demonstrated a positive association (Est.= 0.21, *p* < 0.001). Additionally, study [50] showed that better medication adherence was associated with higher perceived hospital physicians’ communication (B = 0.16, *p* = 0.003). Study [61] found a significant positive correlation between perceived communication and medication adherence (*r* = 0.31, *p* < 0.01). Study [25] reported a statistically significant negative correlation between physicians’ communication and medication adherence (*r* = − 0.29, *p* < 0.001) (higher adherence scores reflected poorer adherence).

#### Primary care physicians

Two high-quality cross-sectional studies [18, 60], one high-quality retrospective cohort study [43], and one moderate-quality cross-sectional study [56] examined the relationship between primary care physicians’ communication and medication adherence. One study [18] found a statistically significant negative correlation between physicians’ empathy score and adherence score (*r* = − 0.40, *p* < 0.001), indicating that higher empathy was associated with improved adherence. Another study [60] found that primary care physicians’ communication was significantly associated with medication adherence (β = 0.38, 95% CI 0.09 to 0.18, *p* < 0.001). Similarly, study [43] showed that better communication from primary care physicians was significantly associated with increased adherence to antihypertensive medications (OR = 1.38, 95% CI 1.14 to 1.67). Study [56] also reported that practitioner communication behaviours were positively associated with better treatment adherence (*r* = 0.41, *p* < 0.01).

#### Nurses

One RCT [47] with some concerns about bias found that communication-based nursing interventions significantly improved adherence among cancer patients.

Supplementary File 4 contains full details of the synthesis by type of continent. Additionally, as a formal subgroup analysis was not feasible, a narrative summary is presented in Supplementary File 5.

## Discussion

This review demonstrates that effective patient–practitioner communication is generally associated with higher treatment adherence, although findings varied by region, practitioner type, and study design. All cross-sectional studies reported significant positive associations between practitioner communication—particularly physicians’ empathy—and medication or overall treatment adherence.

However, findings from RCTs were more mixed. Across six RCTs, the pooled analysis demonstrated a small, non-significant effect in favour of communication interventions (SMD = 0.42, 95% CI − 0.08 to 0.93), with very high heterogeneity (I² = 88%). While one trial, involving nurses in Asian settings, demonstrated significant improvements, pooled analyses—particularly of pharmacist-led interventions conducted in North America— showed a small, non-significant pooled effect. These discrepancies may reflect differences in intervention design, adherence measurement, and methodological quality.

As RCTs are designed to minimise bias through randomisation and controlled conditions, this methodological rigour may result in more conservative effect estimates. Accordingly, the findings suggest that communication interventions alone may have limited effectiveness in some settings and may need to be embedded within broader, multifaceted adherence strategies.

Observational evidence remained more consistent. Eight cross-sectional studies reporting correlation coefficients showed a moderate positive association between practitioner communication and adherence (pooled *r* = 0.35, 95% CI 0.25 to 0.44), although heterogeneity was substantial (I² = 85.5%). Similarly, when restricted to medication adherence outcomes, pooled cross-sectional data demonstrated a statistically significant positive association (pooled *r* = 0.30, 95% CI 0.21 to 0.38), with moderate-to-substantial heterogeneity (I² = 68.4%).

### Comparison with prior reviews

The findings of this review align with earlier reviews examining the relationship between patient–practitioner communication and treatment adherence. An earlier review [2] described adherence as a complex, multifactorial behaviour for which no single factor consistently predicts adherence. A more recent review focusing on communication-based adherence similarly reported heterogeneous and context-dependent effects [71]. These reviews suggest that communication strategies such as patient education, collaborative communication, and counselling approaches can be beneficial in certain clinical settings. However, the existing evidence base is characterised by methodological variability, which limits comparability across studies.

Most notably, the meta-analysis by Zolnierek and DiMatteo (2009) [13] examined physician–patient communication exclusively and reported a significant positive association with adherence, including a 19% higher risk of non-adherence among patients whose physicians communicated poorly. Similar to that review, the present synthesis demonstrates consistent positive associations in observational studies.

However, whereas Zolnierek and DiMatteo (2009) reported statistically significant pooled effects across both observational and intervention studies, the present review identified a small, non-significant pooled effect in RCTs, despite generally positive effect direction. This divergence likely reflects substantial clinical and methodological heterogeneity across trials. Importantly, the prior meta-analysis focused exclusively on physician communication, whereas the present review included multiple practitioner types, potentially introducing greater intervention variability.

The included interventions varied considerably and encompassed motivational interviewing–based interventions, pharmacist-led interventions, and patient–practitioner communication interventions, delivered by different types of healthcare practitioners. In addition, the RCTs enrolled heterogeneous patient populations and employed differing adherence measurement strategies, including self-report questionnaires, pharmacy refill data, and medical chart records. This variability is reflected in the high heterogeneity observed in the pooled analysis (I² = 88%) and wide confidence intervals, which likely reduced statistical power despite generally positive effect directions.

In comparison with RCTs, cross-sectional studies often include larger samples and may have been more susceptible to confounding, potentially explaining their consistently significant positive associations. Together, these findings highlight the influence of study design quality, population characteristics, and heterogeneity on the detection of intervention effects. Nonetheless, the consistent positive associations observed in observational studies highlight that communication remains a key modifiable factor influencing adherence.

### Implications for policy and practice

Our findings highlight the crucial role of effective communication in supporting patient adherence to treatment and care plans. They also highlight the importance of strengthening healthcare practitioners’ communication skills to enhance adherence outcomes. Communication skills training represents a worthwhile investment, but should not be viewed as a standalone solution. Such training is likely to be most effective when tailored to specific clinical contexts and integrated within broader, system-level adherence initiatives.

Researchers can build on our findings to explore and evaluate communication strategies and interventions that promote patient adherence more effectively.

Policymakers should ensure that healthcare practitioners are effective communicators. They should focus on commissioning evidence-based training and education in communication skills to ensure that healthcare professionals develop and maintain positive communication with patients. Such training should be an integral part of medical education, and continue through ongoing professional development. It should equip healthcare professionals with strategies to engage patients in meaningful conversations about their treatment, address barriers to adherence, build trust, and promote active involvement in care.

In clinical practice, healthcare organisations should embed adherence-focused communication into routine care. This may include the use of motivational interviewing, the provision of clear and consistent information, empathic behaviour and the creation of supportive care environments that encourage patients to adhere to recommended treatments. Integrating such strategies within team-based and multidisciplinary models of care may further enhance their effectiveness. Collectively, these approaches have the potential to improve patient outcomes, optimise healthcare resource utilisation, and contribute to broader public health goals.

### Limitations and future research

There were a number of limitations to this review. Poor reporting of key methodological details—such as the type and number of practitioners involved, any communication training provided, the use of appropriate statistical analyses, and the overall quality and reporting adequacy of the included studies—limited our ability to draw robust conclusions from the available data. Additionally, substantial heterogeneity across studies restricted inferences regarding the most effective communication interventions for improving adherence. Furthermore, in several included studies, the type of healthcare practitioner was not specified; therefore, comparability across provider groups could not be fully assured.

Moreover, medication adherence was predominantly assessed using patient self-report measures, which are susceptible to reporting bias. Compared with objective measures such as pharmacy refill records or electronic monitoring, self-report instruments generally exhibit lower specificity and may misclassify non-adherent patients as adherent, reducing the precision of effect estimates and overall internal validity. Although self-report methods are widely used in clinical and population-based research, they may not fully capture actual adherence behaviour, and their use should be interpreted with caution [72, 73].

Future research should incorporate objective or multimethod adherence assessments to improve validity and reduce the risk of reporting bias. High-quality trials are needed to identify which communication strategies are most effective and how their benefits can be maximised across different clinical contexts.

## Conclusion

Effective communication is a fundamental component of patient care and is consistently associated with improved treatment adherence. This review highlights the importance of viewing communication not just as a clinical skill, but as a strategic lever enhancing patient engagement and supporting health system performance.

## Supplementary Information

Below is the link to the electronic supplementary material.

Supplementary Material 1

## Data Availability

Not applicable.

## Abbreviations

RoB2: Cochrane risk-of-bias tool for randomised trials
ILC: International Longevity Centre
JBI: Joanna-Briggs Institute
PRESS: Peer Review of Electronic Search Strategies
PRISMA-P: Preferred Reporting Items for Systematic Reviews and Meta-Analyses Protocols
RCTs: Randomised controlled trials
SMS: Short message service
SMD: Standardised mean difference
WHO: World Health Organisation

## Declarations

### Previous posting

This protocol of this manuscript was published on OSF pre-print https://osf.io/preprints/osf/tsq9p_v2.
