# Supplementary material for: Examining the relationship between healthcare practitioners’ communication and patient adherence to treatment: a systematic review
Source: BMC Health Serv Res. 2026 Mar 25;26:458. doi: 10.1186/s12913-026-14359-8 (PMC13045089; doi:10.1186/s12913-026-14359-8)
Supplement: Supplementary file 1 — Supplementary Material 1 [file 12913_2026_14359_MOESM1_ESM.docx]

**Table of Contents**

[**Supplementary File 1. Definition of Key Terms** 2](#_Toc222401355)

[**Supplementary File 2. Search Strategy** 4](#_Toc222401356)

[Ovid MEDLINE(R) ALL <1946 to January 23, 2025> 4](#_Toc222401357)

[APA PsycInfo 4](#_Toc222401358)

[CENTRAL 5](#_Toc222401359)

[Cinahl 6](#_Toc222401360)

[Scopus 7](#_Toc222401361)

[Numbers of results 25^th^ January 2025 8](#_Toc222401362)

[**Supplementary File 3. PRESS Guideline** 9](#_Toc222401363)

[**Supplementary Figure 1. Flow Diagram of Selection of Studies** 15](#_Toc222401364)

[**Supplementary Figure 2. Meta-analysis of Correlation (r) Studies** 16](#_Toc222401365)

[**Supplementary Table 1. Inclusion and Exclusion Criteria Using PICO and Other Relevant Criteria** 17](#_Toc222401366)

[**Supplementary Table 2. Scales and Tools Used to Measure Communication** 19](#_Toc222401367)

[**Supplementary Table 3. Scales and Tools Used to Measure Adherence** 26](#_Toc222401368)

[**Supplementary Table 4. Risk of Bias Assessment** 31](#_Toc222401369)

[**Supplementary Table 5. GRADE Assessment for Six RCTs** 32](#_Toc222401370)

[**Supplementary Table 6. GRADE Assessment for Adherence to Medication** 33](#_Toc222401371)

[**Supplementary Table 7. GRADE Assessment for Pharmacist Communication Intervention** 34](#_Toc222401372)

[**Supplementary File 4. Synthesis by Study Continent** 35](#_Toc222401373)

[**Supplementary File 5. Sub-group Analysis** 39](#_Toc222401374)

# **Supplementary File 1. Definition of Key Terms**

***Definitions of communication*** ***in this study***

We consider patient (or carer)-practitioner communication to include face-to-face interactions, as well as virtual forms like video calls or phone conversations. We also consider communication specifically aimed at improving adherence, and communication not aimed directly at improving adherence. The former could include cases where practitioners emphasise the importance of following the treatment regimen, providing straightforward instructions, simplifying the regimen, tailoring the treatment in accordance with the patient’s preferences, as well as obtaining support from support networks that could reinforce desirable behaviour, which is also part of the communication process (1). The latter could include counselling and therapeutic empathy, where counselling refers to discussions between patients or carers and healthcare professionals about the importance of intervention, side effects, and other treatment details (2). Empathy is also considered as understanding and expressing understanding, followed by “act[ing] on that understanding ... in a helpful (therapeutic) way” by healthcare practitioners (3).

***Definition of patient adherence*** ***in this study***

We will also use the WHO’s definition of adherence: “the extent to which a person’s behaviour – taking medication, following a diet, and/or executing lifestyle changes, corresponds with agreed recommendations from a healthcare provider” (4). More specifically, we will consider medication adherence as the extent to which the patient's behaviour aligns with recommendations from the prescriber (5). Medication adherence can also be described as the degree to which a patient’s behaviour corresponds with the prescribed medication regime, including timing, dosage and interval of medication intake (6). Based on these definitions, the term adherence that we use in this study is a broader term, encompassing overall adherence to medical advice and other healthcare interventions. This includes not only taking medications as prescribed but also following other aspects of the treatment plan, such as attending appointments and undergoing necessary tests.

**Supplementary File 2. Search Strategy**

Communication and patient adherence Final search strategies, searches run 25^th^ January 2025

Search saved in Medline, APA PsycInfo, CENTRAL, Cinahl

Had issues with personal account in Scopus so search not saved- search string is recorded below.

Ovid MEDLINE(R) ALL <1946 to January 23, 2025>

Ovid MEDLINE(R) ALL <1946 to January 24, 2025>

1 communication/ 106465

2 communicat*.mp. 572248

3 miscommunicat*.mp. 1311

4 ((Legible or understandable or clear or decipherable) adj2 (note or notes)).mp. 42

5 or/1-4 572860

6 exp "Treatment Adherence and Compliance"/ 289185

7 ((appointment* or schedul* or medication* or treatment* or therap* or patient or intervention*) adj3 (accept* or adher* or complian* or comply* or concordan* or conform* or co-operat* or cooperat* or keep* or persist*)).mp. 282119

8 ((appointment* or schedul* or medication* or treatment* or therap* or patient) adj3 (nonadher* or non adher* or nonaccept* or non accept* or noncompl* or non compl* or nonconcordan* or non concordan* or discont* or abstention* or abstain* or stop* or abandon* or dropout* or drop* out* or cancel*)).mp. 90356

9 6 or 7 or 8 487089

10 5 and 9 28538

11 limit 10 to (meta analysis or "review" or "systematic review") 3691

12 limit 10 to "qualitative (best balance of sensitivity and specificity)" 11693

13 11 or 12 14297

14 10 not 13 14241

15 limit 14 to yr="2014 - 2025" 6720

APA PsycInfo

| **#** | **Query** | **Limiters/Expanders** | **Results** |
| --- | --- | --- | --- |
| S13 | S10 NOT S11 | Limiters - Publication Year: 2014-2025 Expanders - Apply equivalent subjects Search modes - Proximity | 2,030 |
| S12 | S10 NOT S11 | Expanders - Apply equivalent subjects Search modes - Proximity | 4,791 |
| S11 | S5 AND S9 | Limiters - Methodology: LITERATURE REVIEW, -Systematic Review, META ANALYSIS, METASYNTHESIS, QUALITATIVE STUDY Expanders - Apply equivalent subjects Search modes - Proximity | 1,760 |
| S10 | S5 AND S9 | Expanders - Apply equivalent subjects Search modes - Proximity | 6,551 |
| S9 | S6 OR S7 OR S8 | Expanders - Apply equivalent subjects Search modes - Proximity | 91,060 |
| S8 | ((appointment* or schedul* or medication* or treatment* or therap* or patient) N2 (nonadher* or non adher* or nonaccept* or non accept* or noncompl* or non compl* or nonconcordan* or non concordan* or discont* or abstention* or abstain* or stop* or abandon* or dropout* or drop* out* or cancel*)) | Expanders - Apply equivalent subjects Search modes - Proximity | 21,579 |
| S7 | ((appointment* or schedul* or medication* or treatment* or therap* or patient or intervention*) N2 (accept* or adher* or complian* or comply* or concordan* or conform* or co-operat* or cooperat* or keep* or persist*)) | Expanders - Apply equivalent subjects Search modes - Proximity | 76,152 |
| S6 | DE "Treatment Compliance" | Expanders - Apply equivalent subjects Search modes - Proximity | 18,873 |
| S5 | S1 OR S2 OR S3 OR S4 | Expanders - Apply equivalent subjects Search modes - Proximity | 502,208 |
| S4 | ((Legible or understandable or clear or decipherable) N1 (note or notes)) | Expanders - Apply equivalent subjects Search modes - Proximity | 16 |
| S3 | miscommunicat* | Expanders - Apply equivalent subjects Search modes - Proximity | 956 |
| S2 | communicat* | Expanders - Apply equivalent subjects Search modes - Proximity | 489,104 |
| S1 | DE "Communication" OR DE "Communication Skills" OR DE "Language Proficiency" OR DE "Rhetoric" OR DE "Writing Skills" | Expanders - Apply equivalent subjects Search modes - Proximity | 81,678 |

CENTRAL

Date Run: 27/01/2025 03:14:20

Comment: Based on Medline search of same name 24th January 2025

ID Search Hits

#1 MeSH descriptor: [Communication] this term only 3714

#2 communicat*:ti,ab,kw 37041

#3 miscommunicat*:ti,ab,kw 69

#4 ((Legible or understandable or clear or decipherable) NEAR/1 (note or notes)):ti,ab,kw 1

#5 #1 OR #2 OR #3 OR #4 37069

#6 MeSH descriptor: [Treatment Adherence and Compliance] explode all trees 38364

#7 ((appointment* or schedul* or medication* or treatment* or therap* or patient or intervention*) NEAR/2 (accept* or adher* or complian* or comply* or concordan* or conform* or co-operat* or cooperat* or keep* or persist*)):ti,ab,kw 65642

#8 ((appointment* or schedul* or medication* or treatment* or therap* or patient) NEAR/2 (nonadher* or non adher* or nonaccept* or non accept* or noncompl* or non compl* or nonconcordan* or non concordan* or discont* or abstention* or abstain* or stop* or abandon* or dropout* or drop* out* or cancel*)):ti,ab,kw 431454

#9 #6 OR #7 OR #8 446990

#10 #5 AND #9 8905 – of which 8770 are trials, when limited to 2014 -date, 6296

Limited by date. Registry entries retained.

Cinahl

| **#** | **Query** | **Limiters/Expanders** | **Results** |
| --- | --- | --- | --- |
| S15 | S11 NOT S14 | Limiters - Publication Date: 20140101-20251231 Expanders - Apply equivalent subjects Search modes - Proximity | 3,180 |
| S14 | S12 OR S13 | Limiters - Publication Date: 20140101-20251231 Expanders - Apply equivalent subjects Search modes - Proximity | 1,191 |
| S13 | S5 AND S9 | Limiters - Publication Date: 20140101-20251231; Clinical Queries: Qualitative - Best Balance Expanders - Apply equivalent subjects Search modes - Proximity | 734 |
| S12 | S5 AND S9 | Limiters - Publication Date: 20140101-20251231; Publication Type: Meta Analysis, Meta Synthesis, Review, Systematic Review Expanders - Apply equivalent subjects Search modes - Proximity | 498 |
| S11 | S5 AND S9 | Limiters - Publication Date: 20140101-20251231 Expanders - Apply equivalent subjects Search modes - Proximity | 4,371 |
| S10 | S5 AND S9 | Expanders - Apply equivalent subjects Search modes - Proximity | 7,391 |
| S9 | S6 OR S7 OR S8 | Expanders - Apply equivalent subjects Search modes - Proximity | 138,877 |
| S8 | ((appointment* or schedul* or medication* or treatment* or therap* or patient) N2 (nonadher* or non adher* or nonaccept* or non accept* or noncompl* or non compl* or nonconcordan* or non concordan* or discont* or abstention* or abstain* or stop* or abandon* or dropout* or drop* out* or cancel*)) | Expanders - Apply equivalent subjects Search modes - Proximity | 29,900 |
| S7 | ((appointment* or schedul* or medication* or treatment* or therap* or patient or intervention*) N2 (accept* or adher* or complian* or comply* or concordan* or conform* or co-operat* or cooperat* or keep* or persist*)) | Expanders - Apply equivalent subjects Search modes - Proximity | 117,264 |
| S6 | MH "Patient Compliance+" | Expanders - Apply equivalent subjects Search modes - Proximity | 60,461 |
| S5 | S1 OR S2 OR S3 OR S4 | Expanders - Apply equivalent subjects Search modes - Proximity | 257,736 |
| S4 | ((Legible or understandable or clear or decipherable) N1 (note or notes)) | Expanders - Apply equivalent subjects Search modes - Proximity | 23 |
| S3 | miscommunicat* | Expanders - Apply equivalent subjects Search modes - Proximity | 696 |
| S2 | communicat* | Expanders - Apply equivalent subjects Search modes - Proximity | 257,451 |
| S1 | MH "Communication" | Expanders - Apply equivalent subjects Search modes - Proximity | 103,917 |

Scopus

(( TITLE-ABS-KEY ( ( appointment* OR schedul* OR medication* OR treatment* OR therap* OR patient ) W/2 ( nonadher* OR "non adher*" OR nonaccept* OR "non accept*" OR noncompl* OR "non compl*" OR nonconcordan* OR "non concordan*" OR discont* OR abstention* OR abstain* OR stop* OR abandon* OR dropout* OR "drop* out*" OR cancel* ) ) ) OR ( TITLE-ABS-KEY ( ( appointment* OR schedul* OR medication* OR treatment* OR therap* OR patient OR intervention* ) W/2 ( accept* OR adher* OR complian* OR comply* OR concordan* OR conform* OR co-operat* OR cooperat* OR keep* OR persist* ) ) )) AND (TITLE-ABS-KEY ( communicat* OR miscommunicat* OR ( ( legible OR understandable OR clear OR decipherable ) W/1 ( note OR notes ) ) )) AND PUBYEAR > 2013 AND PUBYEAR < 2026 AND ( EXCLUDE ( DOCTYPE,"re" ) OR EXCLUDE ( DOCTYPE,"cp" ) OR EXCLUDE ( DOCTYPE,"ch" ) OR EXCLUDE ( DOCTYPE,"cr" ) OR EXCLUDE ( DOCTYPE,"bk" ) )

ANDNOT

TITLE-ABS-KEY ( "Empirical Research" OR interview OR "Interviews as Topic" OR "Personal Narratives" OR "Focus Groups" OR narration OR "Nursing Methodology Research" OR "Narrative Medicine" OR interview* OR qualitative OR theme* OR thematic OR "ethnological research" OR ethnograph* OR ethnomedicine OR ethnonursing OR phenomenol* OR "grounded theor*" OR "grounded study" OR "grounded studies" OR "grounded research" OR "grounded analysis" OR "grounded analyses" OR "life stor*" OR emic OR etic OR hermeneutic* OR heuristic* OR semiotic* OR "data saturat*" OR "participant observ*" OR "social construct*" OR postmodern* OR post-structural* OR "post structural*" OR poststructural* OR "post modern*" OR post-modern* OR feminis* OR "action research" OR "cooperative inquir*" OR "co operative inquir*" OR humanistic OR existential OR experiential OR paradigm* OR "field study" OR "field studies" OR "field research" OR "field work" OR "human science" OR "social science" OR "biographical method" OR "theoretical sampl*" OR ( purpos* W/3 sampl* ) OR ( focus W/3 group* ) OR open-ended OR narrative* OR textual OR texts OR "semi-structured" OR "life world*" OR "life-world*" OR "conversation analysis" OR "personal experience*" OR "theoretical saturation" OR "conversation analyses" OR "lived experience" OR "life experience*" OR "cluster sampl*" OR "observational method*" OR "content analysis" OR "constant comparative" OR "constant comparison" OR ( discourse* W/3 analysis ) OR ( discourse* W/3 analyses ) OR ( discurs* W/3 analysis ) OR ( discurs* W/3 analyses ) OR heidegger* OR colaizzi* OR spiegelberg* OR merleau* OR husserl* OR foucault* OR ricoeur OR glaser* OR "van manen*" OR "van kaam*" OR ( corbin* W/2 strauss* ) )

(CADTH Qualitative Studies - Scopus filter retrieved 27^th^ January 2025 from https://searchfilters.cda-amc.ca/list?q=&p=1&ps=20&setName_facet=scopus%20000000%7CScopus&topic_facet=qualitative%20studies%20000000%7CQualitative%20studies

Numbers of results 25^th^ January 2025

| Medline | 6720 |
| --- | --- |
| Cinahl | 3180 |
| Scopus | 6930 |
| Central | 6296 |
| APA PsycInfo | 2030 |
| Total unduplicated | 25156 |

**Supplementary File 3. PRESS Guideline**

***PRESS Guideline* — Search Submission & Peer Review Assessment**

**SEARCH SUBMISSION: THIS SECTION TO BE FILLED IN BY THE SEARCHER**

| Searcher: Keith Nockels | Email: |  |
| --- | --- | --- |
| Date submitted: 23^rd^ January 2025 | Date requested by: | *[Maximum = 5 working days]* |

**Systematic Review Title:**

Examining the relationship between healthcare practitioner's communication and patient adherence to treatment: A protocol for systematic review.

This search strategy is …

| X | My PRIMARY (core) database strategy — First time submitting a strategy for search question and database |
| --- | --- |
|  | My PRIMARY (core) strategy — Follow-up review NOT the first time submitting a strategy for search question and database. If this is a response to peer review, itemize the changes made to the review suggestions |
|  | SECONDARY search strategy— First time submitting a strategy for search question and database |
|  | SECONDARY search strategy — NOT the first time submitting a strategy for search question and database. If  this is a response to peer review, itemize the changes made to the review suggestions |

**Database**

(i.e., MEDLINE,CINAHL…): *[mandatory]*

Medline. Strategies for other databases will be based on the Medline one.

**Interface**

(i.e., Ovid, EBSCO…): *[mandatory]*

Ovid

**Research Question**

(Describe the purpose of the search) *[mandatory]*

This study aims to explore the relationship between patient-practitioner communication and subsequent patient adherence to healthcare intervention.

Studies published in English that quantified the effects of patient-practitioner communication on patient adherence to healthcare intervention.

We are not looking for studies about interprofessional communication, but are looking for studies that describe or discuss face to face communication, including face to face communication using phone or video. We are excluding the use of text messaging, recorded reminders, leaflets and written instructions.

We will use the WHO’s definition of adherence: “the extent to which a person’s behaviour – taking medication, following a diet, and/or executing lifestyle changes, corresponds with agreed recommendations from a healthcare provider”.

**PICO Format**(Outline the PICOs for your question — i.e., Patient, Intervention, Comparison, Outcome, and Study Design — as applicable)

| **P** | Patients |
| --- | --- |
| **I** | Communication with a carer or practitioner |
| **C** |  |
| **O** | Adherence to treatment, appointment arrangements, medication |
| **S** |  |

**Inclusion Criteria**

(List criteria such as age groups, study designs, etc., to be included) *[optional]*

Any age group, any setting, any quantitative study design

**Exclusion Criteria**

(List criteria such as study designs, date limits, etc., to be excluded) *[optional]*

We are excluding qualitative studies, and review articles of any type.

**Was a search filter applied?** No. We have used Ovid limits and clinical queries to identify qualitative studies and reviews, and others will be removed at screening.

**If YES, which one(s) (e.g., Cochrane RCT filter, PubMed Clinical Queries filter)? Provide the source if this is a published filter.** *[mandatory if YES to previous question* — *textbox]*

Other notes or comments you feel would be useful for the peer reviewer? ***[optional]***

Please copy and paste your search strategy here, exactly as run, including the number of hits per line. ***[mandatory]***

**Ovid MEDLINE(R) ALL <1946 to January 22, 2025>**

1 communication/ 106425

2 communicat*.mp. 571931

3 miscommunicat*.mp. 1310

4 ((Legible or understandable or clear or decipherable) adj2 (note or notes)).mp. 42

5 or/1-4 572542

6 exp "Treatment Adherence and Compliance"/ 289011

7 ((appointment* or schedul* or medication* or treatment* or therap* or patient or intervention*) adj3 (accept* or adher* or complian* or comply* or concordan* or conform* or co-operat* or cooperat* or keep* or persist*)).mp. 281908

8 ((appointment* or schedul* or medication* or treatment* or therap* or patient) adj3 (nonadher* or non adher* or nonaccept* or non accept* or noncompl* or non compl* or nonconcordan* or non concordan* or discont* or abstention* or abstain* or stop* or abandon* or dropout* or drop* out* or cancel*)).mp. 90290

9 6 or 7 or 8 486737

10 5 and 9 28506

11 limit 10 to (meta analysis or "review" or "systematic review") 3685

12 limit 10 to "qualitative (best balance of sensitivity and specificity)" 11678

13 11 or 12 14278

14 10 not 13 14228

15 limit 14 to yr="2014 - 2025" 6707

**(Add more space, as necessary.)**

**PEER REVIEW ASSESSMENT: THIS SECTION TO BE FILLED IN BY THE REVIEWER**

|  | Reviewer: UHL Clinical Librarian team | Email: [pip.divall@uhl-tr.nhs.uk](mailto:pip.divall@uhl-tr.nhs.uk) (service manager) | Date completed: 23^rd^ January 2025 | | |
| --- | --- | --- | --- | --- | --- |
|  |  |  |  | | |
|  | **1. TRANSLATION** |  |  | | |
| A -­‐No revisions | | ☐ |  |  |  |
| B -­‐ Revision(s) suggested | | ☐ |  |  |  |
| C -­‐ Revision(s) required | | ☐ |  |  |  |

If “B” or “C,” please provide an explanation or example:

Not asked about translation of strategy

**2. BOOLEAN AND PROXIMITY OPERATORS**

| A -­‐No revisions | X |
| --- | --- |
| B -­‐ Revision(s) suggested | ☐ |
| C -­‐ Revision(s) required | ☐ |

If “B” or “C,” please provide an explanation or example:

**3. SUBJECT HEADINGS**

| A -­‐No revisions | ☐ |
| --- | --- |
| B -­‐ Revision(s) suggested | X |
| C -­‐ Revision(s) required | ☐ |

If “B” or “C,” please provide an explanation or example:

Does it maybe need a separate concept for clinician-patient relationship and some of the free-text and MeSH terms that come with that? So ^1^clinican-patient relationship AND communication AND treatment adherence? Would help make it more focussed.

**4. TEXT WORD SEARCHING**

| A -­‐No revisions | ☐ |
| --- | --- |
| B -­‐ Revision(s)suggested | X |
| C -­‐ Revision(s) required | ☐ |

If “B” or “C,” please provide an explanation or example:

I did a quick search to see if .ti,ab,kw,kf. Was better than .mp. And it brought back MORE results, which seemed all kinds of wrong.

A couple of things jump out at me. If it's just face to face communication they're after, the current search will bring up a lot of irrelevant results. I imagine their exclusion criteria will help whittle these down but it's probably better to articulate this sooner rather than later in the strategy. Can they be more specific about the face to face stuff.

I agree that some of the terms are very broad. I wonder if the search might be slightly more focussed if line 2 was searched in only ti and kf? 'Communication' a very broad term.

The brief says no written communication, so line 4 perhaps take out note/notes in favour of point 5's suggestions.

I'm not sure what is best practice for excluding certain study types. I would be tempted not to do it at all and refine the terms more to manage the numbers. But, if this method were to be used, perhaps using the specificity-maximising filters would be best, to reduce the risk of excluding anything potentially relevant.

The remit seems to be face to face communication and yet it has lines in there regarding written communication. This could be tightened up with more specifics (verbal communication, etc.) rather than using the general term communication. I'm sure there must be more defined terms under the MeSH term 'communication', although I haven't looked.

There are also MeSH terms about profession-patient interrelations, which would be more on target and would cover all the healthcare practitioners. Although, they then may need to narrow this if it brings back too much.

So I think they need more help defining their themes.

Line 8 has too much in it. For example 'schedule' as a term will just bring too much back. I think they need to stick to the major elements of treatment adherence and compliance, as may be determined by the MeSH. I'm sure there are additions but it all seems a bit too broad to me.

I am wondering if line 4 (or a new line) should include more on the patient/practitioner communication ‘in person’? So something like..

((Legible or understand* or clear or decipher*) adj2 (note or notes or consult* or appointment*)).mp.

**5. SPELLING, SYNTAX, AND LINE NUMBERS**

| A -­‐No revisions | X |
| --- | --- |
| B -­‐ Revision(s)suggested | ☐ |
| C -­‐ Revision(s) required | ☐ |

If “B” or “C,” please provide an explanation or example:

**6. LIMITS AND FILTERS**

| A -­‐No revisions | X |
| --- | --- |
| B -­‐ Revision(s) suggested | ☐ |
| C -­‐ Revision(s) required | ☐ |

If “B” or “C,” please provide an explanation or example:

OVERALL EVALUATION (Note: If one or more “revision required” is noted above, the response below must be “revisions required”.)

| A -­‐No revisions | ☐ |
| --- | --- |
| B -­‐ Revision(s) suggested | X |
| C -­‐ Revision(s) required | ☐ |

Additional comments: N/A

# **Supplementary Figure 1. Flow Diagram of Selection of Studies**

**Supplementary Figure 2. Meta-analysis of Correlation (r) Studies**


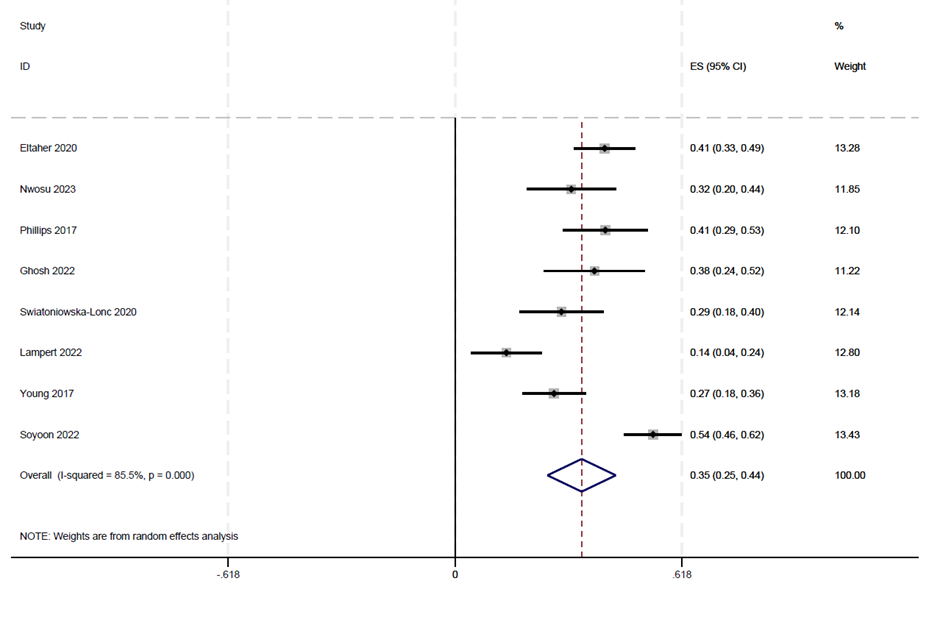


**Supplementary Table 1. Inclusion and Exclusion Criteria Using PICO and Other Relevant Criteria**

| **Element** | **Inclusion** | **Exclusion** |
| --- | --- | --- |
| **Study design** | Any study that quantitatively reported a relationship between patient-practitioner communication intervention and patient adherence to healthcare intervention.  We will include experimental studies where patient-practitioner communication was an intervention (and adherence was an outcome), as well as observational studies that measured patient-practitioner communication and its association with adherence, for example, in a cross-sectional study. | Systematic reviews  Narrative reviews  Full-text not available  Study protocols |
| **Population-participants** | Patients with both chronic and acute conditions or carers in any setting, as long as the communication is between the patient (or carer) and practitioner. | Healthy volunteers, simulated patients. |
| **Population-practitioners** | Healthcare practitioners (any). |  |
| **Intervention** | Patient-practitioner communication. This will include live conversations, encompassing both face-to-face communication and communication via digital media (including telephone, mobile phone, and video) between healthcare practitioners and patients or carers. We will include patient-practitioner aimed specifically at improving adherence as well as general patient-practitioner communication that is not specifically aimed at improving adherence (such as counselling and empathic behaviour). | Electronic reminders, recorded messages and voicemails, emails, short message service (SMS) reminders, written instructions and leaflets. |
| **Comparison** | For studies that have a control group: the comparator is usual care or a less comprehensive communication approach provided as part of standard care.  For studies that do not have a control group: n/a. |  |
| **Outcomes** | Patient adherence to healthcare intervention. | Studies that do not include and measure patient adherence as an outcome. |
| **Language** | Any | - |
| **Timing of intervention** | Any | - |

# **Supplementary Table 2. Scales and Tools Used to Measure Communication**

| **Study** | **Name of scale or tool** | **Scale items** |
| --- | --- | --- |
| Mosleh Ibrahim 2020 | Consultation and Relational Empathy (CARE) measure | Making you feel at ease (being friendly and warm towards you, treating you with respect; not cold or abrupt).  Letting you tell your story (giving you time to fully describe your problem in your own words; not interrupting or diverting you).  Really listening (paying close attention to what you were saying; not looking at the notes or computer as you were talking).  Being interested in you as a whole person (asking or knowing relevant details about your life, your situation, not treating you as “just a number”).  Fully understanding your concerns (communicating that they had accurately understood your concerns; not overlooking or dismissing anything).  Showing care and compassion (seeming genuinely concerned, supportive, and caring; not being indifferent or “detached”).  Being positive (having a positive approach and attitude; being honest but not negative about your problems).  Explaining things clearly (fully answering your questions; explaining clearly; giving you adequate information; not being vague).  Helping you to take control (exploring with you what you can do to improve your health yourself; encouraging rather than “lecturing” you).  Making a plan of action with you (discussing the options, involving you in decisions as much as you want to be; not making decisions for you without you). |
| Gonderen Cakmak 2021 | Not reported (NR) | NR |
| Vinluan 2015 | NR | NR |
| Lee 2017 | Communication subscale | Thoroughness of your doctor s questions about your symptoms and how you are feeling.  Attention your doctor gives to what you have to say.  Doctor s explanations of your health problems or treatments that you need.  Doctor s instructions about symptoms to report and when to seek further care.  Doctor s advice and help in making decisions about your care.  How often do you leave your doctor s office with unanswered questions? |
| Eltaher 2020 | Consultation and Relational Empathy (CARE) Measure | See above. |
| Fosu 2022 | Communication Assessment Tool | Greeted me in a way that made me feel comfortable  Treated me with respect.  Showed interest in my ideas about my health.  Understood my main health concerns.  Paid attention to me (looked at me, listened).  Let me talk without interruptions.  Gave me as much information as I wanted.  Talked in terms I could understand.  Checked to be sure I understood everything.  Encouraged me to ask questions.  Involved me in decisions as much as I wanted.  Discussed next steps.  Showed care and concern.  Spent the right amount of time with me.  Staff treated me with respect. |
| Burmeister 2023 | Patient Cantered Communication | The doctor did not fully discuss with the patient what was causing the patient's problem.  The doctor showed a genuine interest in the patient's health.  The doctor encouraged the patient to express concerns and worries.  The doctor made the patient feel completely at ease during the consultation.  The doctor did not treat the patient as an equal in this consultation.  The doctor thoroughly explained everything to the patient.  The doctor asked for the patient's opinion about what to do about his/her condition.  The doctor was very informative about the patient health.  The doctor asked for the patient's thoughts about his/her health.  The doctor's explanations and recommendations were clear and easy to understand.  The doctor tried to reassure and comfort the patient.  The doctor seemed to care about the patient's feelings. |
| Teymuri 2023 | Burton physician-patient communication scale | Making you feel at ease: (Being friendly and warm towards you, treating you with respect; not cold or abrupt)  Letting you tell "your" story: (Giving you time to fully describe your illness in your own words; not interrupting or diverting you)  Really listening: (Paying close attention to what you were saying; not looking at the notes or computers as you were talking)  Being interested in you as a whole person: (Asking/knowing relevant details about your life, your situation; not treating you as "just a number")  Fully understanding your concerns: (Communicating that he/she had accurately understood your concerns; not overlooking or dismissing anything)  Showing care and compassion: (Seeming genuinely concerned, connecting with you on a human level; not being indifferent or "detached")  Being positive: (Having a positive approach and a positive attitude; being honest but not negative about your problems)  Explaining things clearly: (Fully answering your questions, explaining clearly, giving you adequate information; not being vague)  Helping you to take control: (Explaining with you what you can do to improve your health yourself; encouraging rather than "lecturing" you)  Making a plan of action with you: (Discussing the options, involving you in decisions as much as you want to be involved; not ignoring your views)  How would you rate your consultation with this doctor today? (General assessment of the physician-patient interaction) |
| Nwosu 2023 | Doctor Patient Communication Behaviour Scale (based on the Four Habits Model) | The doctor ignores my concerns.  The doctor clearly explained the medical condition.  The doctor gave opportunities to ask questions.  I could give my opinion.  The doctor used many technical terms.  The doctor provided a lot of information.  The doctor provided comfortable treatment.  Checked that I understood everything |
| Eyler 2016 | NR | NR |
| Michiels 2019 | NR | NR |
| Swain 2015 | Health Communication Checklist (HCC) | NR |
| Lampert 2022 | The Communication About Medication by Providers an Adolescent and Young Adult Version (CAMP-AYA) | The provider clearly explained why it was important to follow the directions and what effects there might be if I did not follow directions.  The provider clearly told me what side effects I might expect from the medication.  The provider gave me suggestions for how I could remember to take the medication. |
| Ocansey 2024 | Doctor-Patient Communication (DPC-13) | Did the doctor listen to you carefully during the consultation?  Did the doctor allow you to talk without interrupting you?  Did the doctor encourage you to express yourself / talk?  Did the doctor examine you thoroughly?  Do you feel that the doctor understood you?  Was it easy to understand what the doctor said?  Do you feel you were given all the necessary information?  Did the doctor explain the advantages and disadvantages of the treatment or care strategy?  Did the doctor involve you in the decision-making?  In your opinion, did the doctor have a reassuring attitude and way of talking?  Did the doctor make sure that you understood his explanations and instructions?  Do you have confidence in this doctor?  Did the doctor reply to all your expectations and concerns? |
| Young 2017 | A scale that assessed respondent’s perceptions about the quality of their interactions with asthma care providers. | gave you a chance to ask all the health-related questions you had;  gave the attention you needed to your feelings and emotions;  helped you deal with feelings of uncertainty about your health and health care;  how often did you feel you could rely on doctors, nurses or other health professionals to take care of your health care needs;  involved you in decisions about your health care as much as you wanted;  made sure you understood the things you needed to do to take care of your health. |
| Phillips 2017 | CS-SRM communication scale | My primary care provider discussed with me which symptoms are related to my medically unexplained symptom (MUS) and which are not.  My primary care provider discussed with me what might be the cause of my MUS.  My primary care provider discussed with me why my symptoms are medically unexplained.  My primary care provider told me what they were looking for during the physical exam.  My primary care provider told me how long I could expect to have this problem/MUS.  My primary care provider gave me clear instructions about my treatment for my MUS: what to do, when, how often, and for how long.  My primary care provider told me what I might expect for my treatment for my MUS.  My primary care provider gave me some tips to help me work my MUS-treatment into my daily routine.  My primary care provider told me how to monitor my problem to see if the MUS-treatment is working.  My primary care provider discussed with me whether or not time would resolve my MUS.  My primary care provider discussed with me whether or not stress might be playing a role in my MUS.  My primary care provider discussed with me that the initial cause of my MUS may differ from what is causing them to continue. |
| Swiatoniowska-Lonc 2020 | Communication Assessment Tool | The physician  Greeted me in a way that made me feel comfortable.  Treated me with respect.  Showed interest in my ideas about my health.  Understood my main health concerns.  Paid attention to me (looked at me, listened carefully).  Let me talk without interruptions.  Gave me as much information as I wanted.  Talked in terms I could understand.  Checked to be sure I understood everything.  Encouraged me to ask questions.  Involved me in decisions as much as I wanted.  Discussed next steps, including any follow-up plans.  Showed care and concern.  Spent the right amount of time with me. |
| Chang 2021 | Consumer Assessment of Healthcare Providers and Systems (CAHPS) | How often does the care provider  listen carefully to the patient;  explain to the patient;  show respect to the patient;  and spend enough time with the patient? |
| Soyoon 2022 | Health Care Climate Questionnaire (HCCQ). | I feel that my physician has provided me choices and options.  I feel understood by my physician.  I am able to be open with my physician at our meetings.  My physician conveys confidence in my ability to make changes.  I feel that my physician accepts me.  My physician has made sure I really understand about my condition and what I need to do.  My physician encourages me to ask questions.  I feel a lot of trust in my physician.  My physician answers my questions fully and carefully.  My physician listens to how I would like to do things.  My physician handles people’s emotions very well.  I feel that my physician cares about me as a person.  I don’t feel very good about the way my physician talks to me.  My physician tries to understand how I see things before suggesting a new way to do things.  I feel able to share my feelings with my physician. |
| Du 2020 | CAHPS | How often did your personal doctor explain things in a way that was easy to understand?  How often did your personal doctor listen carefully to you?  How often did your personal doctor show respect for what you had to say?  How often did your personal doctor spend enough time with you? |
| Mercadante 2021 | NR | NR |
| Ghosh 2022 | The Doctor-Patient Communication Questionnaire (DPCQ) | Did the doctor listen to you carefully during the consultation?  Did the doctor allow you to talk without interrupting you?  Did the doctor encourage you to express yourself / talk?  Did the doctor examine you thoroughly?  Do you feel that the doctor understood you?  Was it easy to understand what the doctor said?  Do you feel you were given all the necessary information?  Did the doctor explain the advantages and disadvantages of the treatment or care strategy?  Did the doctor involve you in the decision-making?  In your opinion, did the doctor have a reassuring attitude and way of talking?  Do you think the doctor was in general respectful?  Did the doctor make sure that you understood his explanations and instructions?  Do you think the doctor told the whole truth?  Do you have confidence in this doctor?  Did the doctor reply to all your expectations and concerns? |
| Trepka 2023 | Provider communication scale (from CAHPS) | In the last 12 months, how often did [this provider] show respect for what you had to say?  In the last 12 months, how often did [this provider] spend enough time with you?  In the last 12 months, how often did [this provider] use medical words you did not understand?  4. In the last 12 months, how often did [this provider] talk too fast when talking with you?  5. In the last 12 months, how often did [this provider] listen carefully to you? |
| Barakat 2024 | The Doctor Patient Communication (DPC) scale | Did the doctor listen to you carefully during the consultation?  Did the doctor allow you to talk without interrupting you?  Do you feel that the doctor understood you?  Did the doctor involve you in the decision-making?  Did the doctor reply to all your expectations and concerns? |

# **Supplementary Table 3. Scales and Tools Used to Measure Adherence**

| **Study** | **Name of scale or tool** | **Scale items** | **Measurement approach** |
| --- | --- | --- | --- |
| Ibrahim 2020 | NR | NR | Medical chart records |
| Gonderen Cakmak 2021 | Oral Chemotherapy Adherence Scale (OCAS) | I take my oral chemotherapy medication at the same time every day.  I forget to take my oral chemotherapy medication.  I stop taking my oral chemotherapy medication when I feel better.  I stop taking my oral chemotherapy medication when I feel worse.  I take the correct dose of my oral chemotherapy medication.  I skip doses of my oral chemotherapy medication.  I take my oral chemotherapy medication exactly as prescribed.  I adjust my medication dose on my own.  I take all my oral chemotherapy medication even if I feel sick.  I run out of my oral chemotherapy medication before I can get a refill.  I take my medication even when I am away from home.  I double my dose when I forget to take it the previous time.  I follow my treatment schedule closely.  I discuss any problems with my oral chemotherapy medication with my healthcare provider.  I avoid taking my medication due to side effects.  I take my medication even if I am busy.  I rely on reminders (e.g., alarms, pillboxes) to take my medication.  I believe that taking the medication as prescribed is important.  I understand the instructions for taking my oral chemotherapy medication. | Self-report |
| Vinluan 2015 | Morisky Medication Adherence Scale (MMAS-8) | Do you sometimes forget to take your medications?  People sometimes miss taking their medications for reasons other than forgetting. Thinking over the past two weeks, were there any days when you did not take your medication?  Have you ever cut back or stopped taking your medication without telling your doctor because you felt worse when you took it?  When you travel or leave home, do you sometimes forget to bring along your medication?  Did you take all your medications yesterday?  When you feel like your condition is under control, do you sometimes stop taking your medication?  Taking medication every day is a real inconvenience for some people. Do you ever feel hassled about sticking to your treatment plan?  How often do you have difficulty remembering to take all your medications? | Self-report |
| Lee 2017 | Morisky Medication Adherence Scale | See above. | Self-report |
| Eltaher 2020 | General Medication Adherence Scale (GMAS) | Do you ever forget to take your diabetic medication?  Do you ever have problems remembering to take your diabetic medication?  When you feel better, do you sometimes stop taking your diabetic medication?  Sometimes if you feel worse when you take your diabetic medication, do you stop taking it? | Self-report |
| Fosu 2022 | Medication Adherence Rating Scale | I sometimes forget to take my antiretroviral medicine.  I sometimes stop taking my antiretroviral medicine when I feel worse. | Self-report |
| Burmeister 2023 | NR | NR | Medical chart records |
| Teymuri 2023 | Morisky Medication adherence scale | See above. | Self-report |
| Nwosu 2023 | Medication adherence report scale-5 (MARS-5) | I forgot to take them.  I altered the dose.  I chose to stop taking them for a while.  I missed a dose.  I took less than instructed. | Self-report |
| Eyler 2016 | NR | Were you able to pick up your medication at the pharmacy? (If no, why not?)  If you are able to, would you get your pill bottle and tell me how many pills are left?  How many antibiotic pills would you say that you missed during treatment?  How many times were you late remembering the medication, but took it within 24 hours?  Have you had any side effects from the medication? What are side effects that you should be watching out for?  Does anyone help you with your medications?  What types of obstacles prevented you from being able to take your medication? | Pharmacy verification and self-report |
| Michiels 2019 | Medication Possession Ratio | NR | Pharmacy refill data |
| Swain 2015 | Hypertension Compliance Scale | How often do you forget to take your high blood pressure medicine?  How often do you decide NOT to take your high blood pressure medicine?  How often do you eat salty food?  How often do you shake salt on your food before you eat it?  How often do you eat fast food?  How often do you make the next appointment before you leave the doctor’s office?  How often do you miss scheduled appointments?  How often do you forget to get prescriptions filled?  How often do you run out of high blood pressure pills?  How often do you skip your high blood pressure medicine before you go to the doctor?  How often do you miss taking your high blood pressure pills when you feel better?  How often do you miss taking your high blood pressure pills when you feel sick?  How often do you take someone else high blood pressure pills?  How often do you miss taking your high blood pressure pills when you are careless? | Self-report |
| Lampert 2022 | Visual Analog scale (VAS) Rating | We would be surprised if most people take 100% of their medications. On the slider below, 0% means you have taken none of that medication this past month, 50% means you have taken half of that medication this past month, and 100% means you have taken every single dose this past month. If you have not needed to take that medicine this past month, think back to the most recent time when you had to take it and answer for that. | Self-report |
| Ocansey 2024 | Medication Adherence Report Scale-5 (MARS-5) | I take less than instructed.  I stop taking them for a while.  I miss out a dose.  I alter the dose.  I forget to take them. | Self-report |
| Young 2017 | Morisky Medication Adherence Scale (MMAS) | See above. | Self-report |
| Phillips 2017 | NR | To what degree did you follow your PCPs recommendations for MUS treatment in the past 6 months? | Self-report |
| Swiatoniowska-Lonc 2020 | The Adherence to Refills and Medication Scale (ARMS) | How often do you forget to take your medicine?  How often do you decide not to take your medicine?  How often do you forget to get prescriptions for your medicine?  How often do you run out of medicine?  How often do you skip a dose of your medicine before you go to the doctor?  How often do you miss taking your medicine when you feel better?  How often do you miss taking your medicine when you feel worse?  How often do you miss taking your medicine when you are careless?  How often do you change the dose of your medicine to suit your needs?  How often do you forget to take your medicine when you are supposed to take it more than once a day?  How often do you put off refilling your medicines because they cost too much money?  How often do you plan ahead and refill your medicines before they run out? | Self-report |
| Chang 2021 | NR | NR | Pharmacy refill data |
| Soyoon 2022 | Diabetes Self-Management Questionnaire (DSMQ) | Example items include 'I stick to my diabetes treatment schedule even when I don’t feel well' and 'I continue with my diabetes treatment even if doing so interferes with my daily activities. | Self-report |
| Du 2020 | NR | How often do you forget to take your medicine?  How often do you stop taking medicine when you experience side effects or feel alleviated symptoms? | Self-report |
| Mercadante 2021 | Proportion of days covered (80% being an accepted adherence rate) | NR | Medical chart records |
| Ghosh 2022 | Medication Adherence Rating Scale (MARS) | Do you ever forget to take your medication? Yes/No  Are you careless at times about taking your medicine? Yes/No  When you feel better, do you sometimes stop taking your medicine? Yes/No  Sometimes if you feel worse when you take the medicine, do you stop taking it? Yes/No  I take my medication only when I am sick. Yes/No  It is unnatural for my mind and body to be controlled by medication. Yes/No  My thoughts are clearer on medication. Yes/No  By staying on medication, I can prevent getting sick. Yes/No  I feel weird, like a zombie, on medication. Yes/No  Medication makes me feel tired and sluggish. Yes/No | Self-report |
| Trepka 2023 | NR | In the last 30 days, how good of a job did you do at taking your HIV medication in the way you were supposed to?  In the last 30 days, how often did you take your HIV medication in the way you were supposed?  In the last 30 days, on how many days did you miss at least one dose of any of your HIV medications? | Self-report |
| Barakat 2024 | The Lebanese Medication Adherence Scale | Do you forget to take your medication when you are busy (intensive work or travel?)  Do you forget to take your medication?  Do you get late when it comes to buying your medication packs when they become empty?  Do you stop taking your medication without consulting your doctor if you do not feel better during the treatment period? | Self-report |

# **Supplementary Table 4. Risk of Bias Assessment**

| **Study** | **Study design / Assessment tool** | **Outcome reported** |
| --- | --- | --- |
| Barakat 2024 | Cross-sectional /JBI | High quality |
| Du 2020 | Cross-sectional /JBI | High quality |
| Eltaher 2020 | Cross-sectional /JBI | High quality |
| Chang 2021 | Retrospective cohort study /JBI | High quality |
| Fosu 2022 | Cross-sectional /JBI | High quality |
| Ghosh 2022 | Cross-sectional /JBI | High quality |
| Lampert 2022 | Cross-sectional /JBI | High quality |
| Lee 2017 | Cross-sectional /JBI | High quality |
| Ibrahim 2020 | Cross-sectional /JBI | High quality |
| Nwosu 2023 | Cross-sectional /JBI | High quality |
| Ocansey 2024 | Cross-sectional /JBI | High quality |
| Phillips 2017 | Cross-sectional /JBI | Moderate quality |
| Soyoon 2022 | Cross-sectional /JBI | Moderate quality |
| Swain 2015 | Cross-sectional /JBI | High quality |
| Swiatoniowska-Lonc 2020 | Cross-sectional /JBI | High quality |
| Teymuri 2023 | Cross-sectional /JBI | High quality |
| Trepka 2023 | Cross-sectional /JBI | Moderate quality |
| Young 2017 | Cross-sectional /JBI | High quality |
| Cakmak 2021 | RCT/ ROB2 | Some concerns |
| Eyler 2016 | RCT/ ROB2 | Some concerns |
| Mercadante 2021 | RCT/ ROB2 | High |
| Michiels 2019 | RCT/ ROB2 | Some concerns |
| Burmeister 2023 | RCT/ ROB2 | Some concerns |
| Vinluan 2015 | RCT/ ROB2 | High |

# **Supplementary Table 5. GRADE Assessment for Six RCTs**

| **Certainty assessment** | | | | | | | **№ of patients** | | **Effect** | | **Certainty** | **Importance** |
| --- | --- | --- | --- | --- | --- | --- | --- | --- | --- | --- | --- | --- |
| **№ of studies** | **Study design** | **Risk of bias** | **Inconsistency** | **Indirectness** | **Imprecision** | **Other considerations** | **communication** | **usual care or a less comprehensive communication approach provided as part of standard care** | **Relative (95% CI)** | **Absolute (95% CI)** |  |  |
| **Patient adherence** | | | | | | | | | | | | |
| 6 | randomised trials | serious | serious^b^ | not serious | serious^c^ | none | 372 | 361 | - | SMD **0.42**  (0.08 lower to 0.93 higher) | ⨁◯◯◯ Very low^a,b,c^ | CRITICAL |

**CI:** confidence interval; **SMD:** standardised mean difference

# **Supplementary Table 6. GRADE Assessment for Adherence to Medication**

| **Certainty assessment** | | | | | | | **№ of patients** | | **Effect** | | **Certainty** | **Importance** |
| --- | --- | --- | --- | --- | --- | --- | --- | --- | --- | --- | --- | --- |
| **№ of studies** | **Study design** | **Risk of bias** | **Inconsistency** | **Indirectness** | **Imprecision** | **Other considerations** | **communication** | **usual care or a less comprehensive communication approach provided as part of standard care** | **Relative (95% CI)** | **Absolute (95% CI)** |  |  |
| **Patient adherence** | | | | | | | | | | | | |
| 5 | randomised trials | serious | serious^c^ | not serious^d^ | serious^e^ | none | 349 | 340 | - | SMD **0.53**   (0.05 lower to 1.11 higher) | ⨁◯◯◯ Very low^a,b,c,d,e^ | CRITICAL |

**CI:** confidence interval; **SMD:** standardised mean difference

# **Supplementary Table 7. GRADE Assessment for Pharmacist Communication Intervention**

| **Certainty assessment** | | | | | | | **№ of patients** | | **Effect** | | **Certainty** | **Importance** |
| --- | --- | --- | --- | --- | --- | --- | --- | --- | --- | --- | --- | --- |
| **№ of studies** | **Study design** | **Risk of bias** | **Inconsistency** | **Indirectness** | **Imprecision** | **Other considerations** | **communication** | **usual care or a less comprehensive communication approach provided as part of standard care** | **Relative (95% CI)** | **Absolute (95% CI)** |  |  |
| **Patient adherence** | | | | | | | | | | | | |
| 4 | randomised trials | serious | not serious | not serious | serious^c^ | none | 309 | 300 | - | SMD **0.13**  (0.08 lower to 0.33 higher) | ⨁⨁◯◯ Low^a,b,c^ | CRITICAL |

**CI:** confidence interval; **SMD:** standardised mean difference

# **Supplementary File 4. Synthesis by Study Continent**

The included studies were conducted across several geographic regions: eight studies (7-14) from Asia, eleven (15-25) from North America, two (26, 27) from Europe, and three (28-30) from Africa. A geographic synthesis was undertaken because healthcare delivery models, provider roles, resource availability, cultural norms, and care pathways vary substantially by region and may influence communication practices and patient adherence (31, 32).

Studies conducted in Asia and Africa were predominantly cross-sectional and relied largely on face-to-face patient–practitioner communication and self-reported measures of adherence, with limited use of structured communication interventions or objective adherence monitoring. These regional patterns likely reflect underlying healthcare system characteristics, including differences in healthcare infrastructure, workforce availability, and access to routine follow-up and pharmacy-based data systems.

In contrast, studies from North America more frequently employed structured communication interventions, including pharmacist-led care and telephone-based approaches, and used a wider range of adherence assessment methods, including pharmacy refill data and medical records. Evidence from Europe was limited, with one pharmacy-based RCT using objective adherence monitoring and one cross-sectional study relying on self-reported adherence, limiting conclusions about consistent regional patterns.

***Asia***

Eight cross-sectional studies (7-14) and one RCT were conducted in Asia. All cross-sectional studies were assessed as high-quality (7-11, 13, 14), while the RCT has some concerns regarding risk of bias (12).

Of these, two studies (7, 13) investigated the association between physicians’ empathy, as perceived by diabetic patients, and their adherence to medication (13) and overall treatment (7). One study (7) reported a significant negative correlation between physicians’ empathy and adherence scores (r =–0.40, p<0.001), indicating that higher empathy was associated with improved adherence. The other study (13) found a statistically significant association between physicians’ empathy and overall treatment adherence (OR=1.05, 95% CI 1.0–1.1, p=0.04).

The remaining studies demonstrated a positive association between patient–practitioner communication and adherence. For example, one study (8) found that practitioners’ communication was positively associated with medication adherence (β=0.30, p<0.01). Similarly, another study (9) reported that patient–practitioner communication was positively related to medication adherence (Est.=0.21, p<0.001). Additionally, a study (11) showed that better medication adherence was associated with improved patient–practitioner communication (B=0.16, p<0.001). Another study found (10) a positive correlation between practitioner communication and medication adherence (r=0.38, 95% CI 0.2–0.5, p<0.01). Furthermore, enhanced communication was found to be significantly associated with overall adherence (β=0.2, p<0.01) in study (14).

The RCT (12) also observed that the mean score for medication compliance was significantly higher in the intervention group compared to the control group (p<0.001).

***North America***

Eleven studies (15-25) were conducted in North America. Of these, four were RCT studies (15, 17, 21, 23); two had some concerns about bias (17, 23) and two had high risk of bias (15, 21). Six were cross-sectional studies (16, 18, 19, 22, 24, 25) and one was a retrospective cohort study (20). They were rated as high quality (16, 18-20) and moderate quality (22, 24, 25).

A pooled analysis of three RCT studies (15, 17, 21) found no significant effect of the communication intervention on medication adherence (p=0.8). The certainty of evidence was very low, and the low heterogeneity (I²=3%) suggests that the variation across studies is modest.

Similarly, one RCT study (23) also reported that the patient–practitioner communication improvement did not result in a significant difference in overall adherence to healthcare intervention.

Four cross-sectional studies (18, 19, 24, 25) reporting Pearson correlation coefficients (r) were pooled using a random-effects model. The pooled estimate demonstrated a statistically significant positive association between practitioner communication and adherence (pooled r=0.34, 95% CI 0.16–0.51). Considerable heterogeneity was observed across studies (I²=90.2%, p<0.001).

The remaining three studies (16, 20, 22) reported significant positive associations between patient–provider communication and medication adherence. One study (16) identified patient–practitioner communication as a significant predictor of medication adherence (β=0.25, p<0.001). Similarly, another study (20) reported that higher levels of clinician communication were associated with a 38% increase in the odds of adherence to antihypertensive medications (OR=1.38, 95% CI 1.1–1.6). Additionally, one study (22) reported that adherence was significantly associated with higher patient–provider trust and communication (OR=1.9, 95% CI 1.1–3.4 p=0.01).

***Europe***

Two studies (26, 27), one high -quality cross- sectional (27) and one RCT with some concerns about bias (26) were conducted in Europe. The cross-sectional study (27) found a statistically significant negative correlation between communication and medication adherence (r=−0.20, p<0.001) (higher adherence scores reflected poorer adherence). Similarly, the RCT (26) study demonstrated that enhancing communication between practitioners and patients led to improved medication adherence.

***Africa***

Three high-quality cross-sectional studies (28-30) were conducted in Africa. Effective patient–practitioner communication was found to be significantly and positively associated with medication adherence. One study (28) demonstrated a statistically significant association between patient–practitioner communication and medication adherence (β=0.38, 95% CI 0.0–0.1, p<0.01). Similarly, another study (30) reported a meaningful positive correlation between perceived physician communication and treatment adherence (β=0.45, 95% CI 0.13–0.4, p <0.01). In addition, a study (29) found a significant and positive correlation between perceived communication and medication adherence (r=0.32, p<0.01).

# **Supplementary File 5. Sub-group Analysis**

**Subgroup analysis by type of clinical condition**:

**Chronic conditions**

Chronic conditions were examined in 19 studies: 14 cross-sectional, four RCTs and one retrospective cohort study. These included diabetes (7, 13, 25, 26), hypertension (14, 16, 20, 27), HIV(22, 28), mental disorders (10, 30), heart failure (15), dyslipidaemia (11), respiratory conditions (9, 19), chronic disease cohorts (24) and cancer (12, 23).

Four RCTs (12, 15, 23, 26) evaluated chronic conditions, specifically diabetes, heart failure, and cancer. Pooled analysis demonstrated a moderate, non-significant positive effect of the intervention (SMD=0.54, 95% CI −0.29 to 1.37), with substantial heterogeneity (I² = 91%).

Six correlational studies involving patients with diabetes (7, 25), hypertension (27), mental disorders (10), respiratory conditions (19) and chronic disease cohorts (24) were pooled and suggested a moderate positive association between practitioner communication and adherence (pooled r=0.38, 95% CI 0.30 to 0.47). However, heterogeneity was substantial (I²=68.6%, p=0.007).

The remining cross-sectional studies (9, 11, 13, 14, 16, 22, 28, 30) of chronic conditions consistently reported positive associations between patient–practitioner communication and medication adherence or overall adherence. The single retrospective cohort study (20) in hypertension also supported a positive association between patient–practitioner communication and medication adherence, strengthening trends observed in cross-sectional analyses.

**Acute conditions**

One RCT study (17) evaluate the impact of a pharmacist-led motivational interviewing on antibiotic adherence following discharge in older adults with pneumonia. They study reported that pharmacist-led motivational interviewing had the potential to positively influence antibiotic adherence rates.

**Mixed or unclear population**

Three cross-sectional studies (8, 18, 29) and one RCT (21) included populations in which the condition was not specified or involved multiple conditions.

The three cross-sectional studies (8, 18, 29) consistently reported positive associations between patient–practitioner communication and medication adherence. The RCT (21) suggested that patient adherence behaviour may improve following a telephone call made by pharmacy staff.

**Subgroup analysis by adherence measures**:

**Self-reported adherence**

Among the included studies that used self-reported adherence, 16 were cross-sectional (7-11, 14, 16, 18, 19, 22, 24, 25, 27-30) and two were RCTs (12, 15).

Two RCTs (12, 15), which relied on self-reported adherence, supported a positive effect of communication-based interventions on adherence outcomes. One RCT (12) reported significant improvements in adherence following motivational interviewing delivered via follow-up, whereas another RCT (15) observed short-term improvements in adherence after pharmacist-led discharge counselling, although effects were not sustained at later follow-up points.

Eight cross-sectional studies (7, 10, 18, 19, 24, 25, 27, 29) reporting correlation coefficients (r) and used self-report measures were pooled and suggested a moderate positive association between practitioner communication and adherence (pooled r=0.35, 95% CI 0.25 to 0.44), with substantial heterogeneity (I²=85.5%, p<0.001).

All the remaining cross-sectional studies (8, 9, 11, 14, 16, 22, 28, 30) also reported positive associations.

**Objective measures of adherence**

Six studies assessed adherence used objective measures, including pharmacy refill data, medical chart records and multimethod verification.

Pharmacy refill data: Two studies used pharmacy refill data. One RCT (26) used pharmacy refill data to evaluate adherence in patients with type 2 diabetes. Although adherence was objectively high at baseline (MPR >90%), the pharmacist-delivered information program did not significantly improve adherence compared with usual care, despite improvements in HbA1c.

Similarly, one retrospective cohort study (20) used objective pharmacy refill data found that higher patient–practitioner communication was significantly associated with improved antihypertensive medication adherence.

Medical chart records: Three studies relied on medical chart records, including two RCTs (21, 23) and one cross-sectional study (13).

One RCT (21) found that adherence improved over time in both control and intervention groups, with no significant between-group differences, suggesting that pharmacist contact may influence adherence. Another RCT (23), which evaluated adherence in patients receiving radiation therapy, did not observe significant differences in treatment adherence between intervention and control groups.

The cross-sectional study (13) also used medical records to assess adherence, reporting a positive association between empathic behaviour and adherence to treatment.

Multimethod: One RCT (17) employed a multimethod approach combining pharmacy verification with self-report to assess antibiotic adherence following discharge. The study reported higher adherence rates in the intervention group compared with the control group; however, the difference did not reach statistical significance due to the small sample size. The authors concluded that pharmacist-led motivational interviewing has the potential to positively influence antibiotic adherence during care transitions.

**References**

1. Bukstein DA. Patient adherence and effective communication. Annals of Allergy, Asthma & Immunology. 2016;117(6):613-9.

2. Santo K, Kirkendall S, Laba T-L, Thakkar J, Webster R, Chalmers J, et al. Interventions to improve medication adherence in coronary disease patients: A systematic review and meta-analysis of randomised controlled trials. European journal of preventive cardiology. 2016;23(10):1065-76.

3. Howick J, Bizzari V, Dambha-Miller H. Therapeutic empathy: what it is and what it isn't. Journal of the Royal Society of Medicine. 2018;111(7):233-6.

4. WHO. Adherence to long-term therapies: evidence for action. World Health Organization; 2003. Report No.: 9241545992.

5. Horne R, Weinman J, Barber N, Elliott R, Morgan M, Cribb A, et al. Concordance, adherence and compliance in medicine taking. London: NCCSDO. 2005;2005(40):6.

6. Cramer JA, Roy A, Burrell A, Fairchild CJ, Fuldeore MJ, Ollendorf DA, et al. Medication compliance and persistence: terminology and definitions. Value in health. 2008;11(1):44-7.

7. Eltaher SM, Rashid MA, Mahdy AW, Lotfy AMM. Physicians’ empathy and its effect on adherence to treatment of diabetic patients in Al-Qassim region, Saudi Arabia. Annals of Tropical Medicine and Health. 2020;23(231):638.

8. Teymuri M, Akbarzadeh Pasha A, Yadollahpour M, Gholizadeh Pasha A, Khafri S, Faramarzi M. The Role of Doctor-Patient Communication Skills in Predicting Treatment Adherence. Journal of Babol University of Medical Sciences. 2023;25(1):195-203.

9. Du L, Wu R, Chen X, Xu J, Ji H, Zhou L. Role of treatment adherence, doctor–patient trust, and communication in predicting treatment effects among tuberculosis patients: difference between urban and rural areas. Patient preference and adherence. 2020:2327-36.

10. Ghosh P, Balasundaram S, Sankaran A, Chandrasekaran V, Sarkar S, Choudhury S. Factors associated with medication non-adherence among patients with severe mental disorder-a cross sectional study in a tertiary care centre. Exploratory Research in Clinical and Social Pharmacy. 2022;7:100178.

11. Barakat M, Thiab S, Abdulrazzaq SB, Al-Jamal M, AlHariri F, Bassam Ammari R, et al. Insights into medication adherence among Jordanian patients with dyslipidemia: evaluating health literacy, well-being, and doctor-patient communication. Journal of Pharmaceutical Policy and Practice. 2024;17(1):2410199.

12. Çakmak HSG, Kapucu S, editors. The effect of educational follow-up with the motivational interview technique on self-efficacy and drug adherence in cancer patients using oral chemotherapy treatment: A randomized controlled trial. Seminars in oncology nursing; 2021: Elsevier.

13. Ibrahim HM, Abousada HJ, Alsharif RH, Alshanqiti NM, Alahmadi SM, Alharbi AA. Association of physicians’ empathy with adherence to treatment among diabetic patients at King Fahd hospital, Medina, KSA. World Family Medicine Journal. 2020;18(1):194-202.

14. Swain S, Hariharan M, Rana S, Chivukula U, Thomas M. Doctor-patient communication: impact on adherence and prognosis among patients with primary hypertension. Psychological Studies. 2015;60:25-32.

15. Vinluan CM, Wittman D, Morisky D. Effect of pharmacist discharge counselling on medication adherence in elderly heart failure patients: a pilot study. Journal of Pharmaceutical Health Services Research. 2015;6(2):103-10.

16. Lee W, Noh Y, Kang H, Hong SH. The mediatory role of medication adherence in improving patients’ medication experience through patient–physician communication among older hypertensive patients. Patient preference and adherence. 2017:1119-26.

17. Eyler R, Shvets K, Blakely ML. Motivational interviewing to increase postdischarge antibiotic adherence in older adults with pneumonia. The Consultant Pharmacist®. 2016;31(1):38-43.

18. Lampert SL, Feldman ECH, Durkin LK, Davies WH, Greenley RN. Medication adherence among emerging adults: the influence of provider communication and patient personality. Children's Health Care. 2022;51(1):101-17.

19. Young HN, Len-Rios ME, Brown R, Moreno MM, Cox E. How does patient-provider communication influence adherence to asthma medications? Patient education and counseling. 2017;100(4):696-702.

20. Chang TJ, Bridges JF, Bynum M, Jackson JW, Joseph JJ, Fischer MA, et al. Association between patient‐clinician relationships and adherence to antihypertensive medications among Black adults: an observational study design. Journal of the American Heart Association. 2021;10(14):e019943.

21. Mercadante A, Lee S, Uh K, Chau A, Truong U, Jeong A, et al. Impact of adherence goal awareness intervention on PDC in various settings: Does awareness help modify medication-taking behavior? Exploratory Research in Clinical and Social Pharmacy. 2021;4:100072.

22. Trepka MJ, Ward MK, Fennie K, Sheehan DM, Fernandez SB, Li T, et al. Patient–Provider Relationships and Antiretroviral Therapy Adherence and Durable Viral Suppression Among Women with HIV, Miami-Dade County, Florida, 2021–2022. AIDS patient care and STDs. 2023;37(7):361-72.

23. Burmeister J, Dominello MM, Soulliere R, Baran G, Dess K, Loughery B, et al. A direct patient-provider relationship with the medical physicist reduces anxiety in patients receiving radiation therapy. International Journal of Radiation Oncology* Biology* Physics. 2023;115(1):233-43.

24. Phillips LA, McAndrew L, Laman-Maharg B, Bloeser K. Evaluating challenges for improving medically unexplained symptoms in US military veterans via provider communication. Patient education and counseling. 2017;100(8):1580-7.

25. Soyoon K, Ekaterina M. From compliance to adherence in diabetes self-care: Examining the role of patient’s potential for mindful non-adherence and physician-patient communication. American journal of health promotion. 2022;36(7):1094-103.

26. Michiels Y, Bugnon O, Chicoye A, Dejager S, Moisan C, Allaert F-A, et al. Impact of a community pharmacist-delivered information program on the follow-up of type-2 diabetic patients: a cluster randomized controlled study. Advances in therapy. 2019;36:1291-303.

27. Świątoniowska-Lonc N, Polański J, Tański W, Jankowska-Polańska B. Impact of satisfaction with physician–patient communication on self-care and adherence in patients with hypertension: cross-sectional study. BMC health services research. 2020;20:1-9.

28. Fosu M, Teye-Kwadjo E, Salifu Yendork J. Patient-reported experiences of medication adherence at a community-based HIV clinic, Ghana. Journal of Patient Experience. 2022;9:23743735221107263.

29. Nwosu LC, Edo GI. Mediating role of patient trust in the impact of perceived physician communication on treatment adherence and its implication in healthcare industry. Science, Engineering and Health Studies. 2023:23050026-.

30. Ocansey G, Teye-Kwadjo E, Osafo J. Medication beliefs and adherence to antipsychotic medication in patients diagnosed with schizophrenia: The moderating role of doctor–patient communication. International Journal of Mental Health. 2025;54(1):80-101.

31. Srivastava D, Lafortune G, Paris V, Belloni A. Geographic variations in health care. What do we know and what can be done to improve health system performance?2014.

32. Alkhamees M, Alasqah I. Patient-physician communication in intercultural settings: An integrative review. Heliyon. 2023;9(12):e22667.
